# Biomarker-based assessment of tobacco smoke exposure in Saudi females via analysis of urinary cotinine level

**DOI:** 10.1371/journal.pone.0355166

**Published:** 2026-08-11

**Authors:** Alaa Abdulelah Alansari, Haya I. Aljohar, Ghadeer Alotaibi, Nouf AL-Tuwayr, Muneerah Essa Alsuwaidan, Khetam Mosfer Alotaibi, Hayat Shaibah, Sultan M. Alghadeer, Hala Aldahshan, Afnan Bakhsh, Bandar Alghanem, Samyah Alanazi, Sana Saeed Alqarni

**Affiliations:** 1 Clinical Laboratory Sciences Department, College of Applied Medical Sciences, King Saud University, Riyad, Saudi Arabia; 2 Department of Pharmaceutical Chemistry, College of Pharmacy, King Saud University, Riyadh, Saudi Arabia; 3 Department of Analytical Chemistry, Central Research Laboratory, King Saud University, Riyadh, Saudi Arabia; 4 Department of Medical Laboratories and Blood Bank, King Khalid University Hospital, King Saud University Medical City, Riyadh, Saudi Arabia; 5 Department of Medical Research Core Facility and Platforms, King Abdullah International Medical Research Center, King Saud bin Abdulaziz University for Health Sciences, Ministry of National Guard Health Affairs (MNGHA), Riyadh, Saudi Arabia; 6 Department of Clinical Pharmacy, College of Pharmacy, King Saud University, Riyad, Saudi Arabia; Federal University of Paraiba, BRAZIL

## Abstract

Tobacco smoking remains a leading preventable cause of morbidity and mortality worldwide. In Saudi Arabia, cultural stigma and reliance on self-reported data obstruct precise assessment of female smoking prevalence and second-hand smoke exposure. Urine cotinine, a stable metabolite of nicotine is a well-established objective biomarker for assessing both active and passive tobacco exposure. The current study aimed to: (1) assess tobacco smoke exposure among Saudi females by combining questionnaire data with biomarker measurements, (2) validate an LC-MS/MS method for quantifying urinary cotinine and (3) establish population-specific cutoff values to distinguish smokers and nonsmokers (4) analyse smoking behaviours and influencing factors via a survey questionnaire. A cross-sectional design was employed involving 133 Saudi female participants recruited between May and December 2024. Each provided a urine sample and completed questionnaire on smoking behaviour and second-hand smoke exposure. Urine samples were processed and analysed by LC-MS/MS. Method validation followed ICH-M10 guidelines, assessing linearity (2–500 ng/mL), accuracy (90–107%) and precision (CV < 10%). The method exhibited excellent linearity (R^2^ ≥ 0.9992). Urinary cotinine was detected in 48 of 133 samples, with concentrations ranging from 0.02 to 7.693 ng/mL. Despite 88.7% of participants self-reporting as nonsmokers, 45.9% reported household second-hand smoke exposure. A cutoff value of 7 ng/mL was established to distinguish smokers from nonsmokers based on ROC analysis. Urinary cotinine, quantified by a validated LC-MS/MS method, provides a reliable and objective assessment of tobacco exposure among Saudi females. This approach addresses the limitations of self-reported data and supports the development of targeted public health interventions aimed at reducing active smoking and second-hand smoke exposure in this population.

## Introduction

Tobacco smoking remains a significant public health concern globally, contributing to many preventable diseases and continues to impose a substantial burden on healthcare systems [1]. Smoking prevalence is generally higher among men than women and is often associated with lower educational and socioeconomic status [2]. Most smokers begin smoking during adolescence, with nearly 90% starting between the ages of 15 and 25 [3].

In the Kingdom of Saudi Arabia, tobacco use has become an increasing public health concern, particularly among younger populations [4]. Accurate assessment of smoking prevalence among Saudi females remains challenging because smoking behaviours are frequently underreported due to cultural and social stigma [5,6]. Consequently, reliance solely on self-reported smoking data may underestimate the true prevalence of tobacco use and passive smoke exposure among women [7]. In addition, exposure to second-hand smoke within households remains common among nonsmoking females, particularly through smoking family members [8]. Traditional self-reporting methods are prone to bias and inaccuracies, highlighting the need for reliable and objective biomarkers [9]. These limitations highlight the need for objective and reliable biomarkers capable of accurately assessing tobacco smoke exposure.

Nicotine, the principal addictive alkaloid in tobacco, is rapidly absorbed through the lungs and mucosal tissues and reaches the brain within seconds after smoking [10]. Following absorption, nicotine is extensively metabolized in the liver, with approximately 70–80% converted into cotinine, its primary and more stable metabolite. Cotinine is a well-established biomarker known for its stability and specificity to tobacco exposure, making it a valuable tool for accurate measurement [10].

Due to its longer half-life and high specificity to tobacco exposure, cotinine is widely recognized as a reliable biomarker for evaluating both active and passive smoking. Measurement of urinary cotinine provides an objective alternative to self-reported smoking data and has been increasingly applied in epidemiological and public health research [8,11].

Liquid chromatography-tandem mass spectrometry (LC-MS/MS) is a highly sensitive and specific analytical platform [12], widely used for the quantification of small-molecule metabolites in biological matrices [13,14]. Owing to its high sensitivity, specificity, and rapid data acquisition, LC-MS/MS is well-suited for measuring urinary biomarkers in clinical and public health research [14]. In addition, LC-MS/MS is a leading technique enable the evaluation of secondary metabolism of drugs in blood and urine samples for forensic and therapeutic contexts [15–17]. In tobacco exposure assessment, LC-MS/MS provides reliable quantification of cotinine, the primary metabolite of nicotine, enabling objective evaluation of both active and passive smoking [18,19].

Recent studies continue to advance the application of urinary cotinine as a biomarker for tobacco smoke exposure and refine exposure classification methods [20]. A large multi-cohort study among pregnant women employed receiver operating characteristic (ROC) analysis to establish urinary cotinine thresholds distinguishing active, environmental, and third-hand smoke exposure, emphasizing the importance of population-specific cutoff values [20]. Similarly, a nationwide biomonitoring study in Vietnam applied urinary cotinine analysis to evaluate smoking and second-hand smoke exposure in a low- to middle-income population, demonstrating demographic variability in exposure patterns [21]. Furthermore, recent systematic reviews have highlighted ongoing international efforts to standardize urinary cotinine cutoff concentrations for distinguishing active and passive smoke exposure across different populations [22]. Nevertheless, limited data are available regarding urinary cotinine thresholds among Saudi females, particularly in the context of cultural and social influences affecting smoking disclosure.

Therefore, this study aimed to evaluate urinary cotinine as an objective biomarker for assessing tobacco smoke exposure among Saudi females using a validated LC-MS/MS method. The study further aimed to establish a preliminary population-specific urinary cotinine cutoff value for distinguishing smokers from nonsmokers and to investigate smoking behaviors and second-hand smoke exposure using questionnaire-based assessment. By integrating biochemical verification with self-reported data, this study seeks to improve exposure assessment accuracy and support future tobacco control and public health strategies targeting Saudi women.

## Materials and methods

### Study design

This study was conducted to evaluate urinary cotinine as an objective biomarker for tobacco smoke exposure among Saudi females. The study integrated questionnaire-based assessment with biochemical analysis to compare self-reported smoking behavior with urinary cotinine concentrations. Urine samples were analyzed using a validated liquid chromatography–tandem mass spectrometry (LC-MS/MS) method, followed by statistical analysis to examine associations between cotinine levels, smoking status, and behavioral characteristics.

### Study population and setting

The study was conducted between May and December 2024 in Riyadh, Saudi Arabia. Participants were recruited from multiple settings to include women from different social and occupational backgrounds. Recruitment sites included King Saud Medical City (patients visiting the sample collection department), King Khalid University Hospital (hospital employees), and King Saud University (female students from different colleges). Eligible participants were Saudi females aged 18 years or older who agreed to participate, were able to complete the questionnaire, and provided a urine sample suitable for analysis. Participants who did not complete the questionnaire or whose urine samples could not be matched to questionnaire records were excluded from the final analysis. Although the study population included participants from diverse educational and occupational backgrounds, participant recruitment was conducted using a convenience sampling approach rather than random population-based sampling. Recruitment was facilitated through direct invitation at the participating sites and by providing information about the study objectives and procedures to potential participants.

### Sample size calculation

The minimum required sample size was estimated using the CheckMarket online sample size calculator based on a 95% confidence level and a 5% margin of error, yielding a target sample size of 385 participants [23]. This calculation was based on the estimated Saudi female population reported by the Saudi General Authority for Statistics in 2024 (approximately 9.78 million females) [24]. Recruitment was initiated with the intention of approaching the calculated sample size; however, practical and cultural challenges related to biological sample collection limited the final achievable sample size. A total of 149 participants initially completed the questionnaire, but after applying eligibility criteria and confirming accurate matching between questionnaires and urine samples, 133 participants were included in the final analysis. Therefore, the study should be considered exploratory in nature, and the findings require validation in larger and more representative cohorts.

### Ethical considerations

This study was conducted in accordance with the principles of the Declaration of Helsinki and approved by the Institutional Review Board (IRB) of the College of Medicine, King Saud University, Riyadh, Saudi Arabia, on February 18, 2024 (Reference No. E-24-8587). Eligible participants (Saudi females aged 18 years or older) received a detailed explanation of the study objectives, procedures, potential risks, anticipated benefits, and confidentiality measures before participation. The participant information sheet and consent form were provided in Arabic to ensure full comprehension. Electronic informed consent was obtained from all participants prior to completion of the questionnaire and urine sample collection. Participation was entirely voluntary, and participants were informed of their right to withdraw at any stage without providing a reason or facing any consequences. To ensure confidentiality, each participant was assigned a unique study identification code, and no personally identifiable information was linked to questionnaire responses or laboratory results. All data were securely stored and accessible only to the research team. The study involved minimal risk because it included only non-invasive urine sample collection and a self-administered questionnaire. Although participants did not receive direct personal benefits, their participation contributes to improving the understanding of tobacco exposure and smoking behaviours among Saudi females.

### Questionnaire design and content

Data were collected using a self-administered electronic questionnaire specifically developed according to the objectives of the study and based on previously published literature related to smoking behaviour and tobacco exposure assessment. The questionnaire was reviewed for clarity and content appropriateness prior to implementation; however, formal psychometric validation was not performed. The questionnaire consists of nine sections. **Section 1**, provided an explanation of the study objectives and procedures, followed by the informed consent statement. Participants were required to provide consent electronically before proceeding to the questionnaire or urine sample collection. Each participant was assigned a unique identification number to enable linkage between questionnaire responses and urine samples while maintaining confidentiality. **Section 2**, collected demographic information, including age, educational level, employment status, monthly income, and place of residence. **Section 3** assessed current smoking status using a yes/no response format. **Section 4**, directed only to those who answered “yes” in the previous section, explores the participant’s smoking history. It asks whether the participant considers herself a current or former smoker, the age at which she began smoking, the reasons behind initiating smoking (e.g., peer pressure, curiosity, social acceptance, media influence, etc.), and the methods of smoking used (such as cigarettes, cigars, vapes, shisha, or other). **Section 5**, evaluated smoking frequency and duration, including smoking frequency categories (daily, occasional, or rare smoking) and duration of smoking history. **Section 6**, assessed smoking cessation attempts, including the number of quit attempts, methods used for cessation, motivation to quit smoking, and reasons for cessation attempts. Participants are asked whether they have ever tried to quit smoking, how many times they have attempted to quit, the methods used (such as nicotine replacement therapy), their motivation level on a scale of 1–10, and the reasons for quitting (e.g., health concerns, financial reasons, family relationships, pregnancy, etc.). **Section 7** investigated exposure to second-hand smoke among nonsmokers by assessing household or environmental exposure to smokers and the relationship to smoking individuals (e.g., parents, siblings, husband, or colleagues). Participants reporting regular exposure to smokers despite not actively smoking were classified as passive smokers for analytical purposes.. **Section 8** assessed smoking-related health complaints, including respiratory and cardiovascular symptoms. **Section 9** included an open-ended question, giving participants the opportunity to share any additional comments about their smoking habits or efforts to quit.

### Classification of smoking status

Participants were categorized into three groups based on their responses: smokers, passive smokers (exposed to second-hand smoke), and nonsmokers. These classifications were used as the basis for comparing reported behavior against measured cotinine levels.

### Recruitment and sampling technique

Participants were recruited using convenience sampling from the selected recruitment sites in Riyadh. Potential participants were approached directly by the research team and provided with information regarding the study objectives, procedures, confidentiality measures, and voluntary nature of participation. Women who agreed to participate completed the electronic questionnaire and subsequently provided urine samples for cotinine analysis. To ensure confidentiality and reduce reporting bias, questionnaires were completed privately, and all samples and responses were anonymized using coded identifiers. Participation was entirely voluntary, and participants were informed that they could withdraw from the study at any stage without consequences.

### Urine sample collection and handling

Midstream urine samples were collected from participants in sterile urine containers and transported to the analytical laboratory for processing and cotinine quantification. Sample preparation, extraction procedures, LC-MS/MS analytical conditions, and method validation were conducted at the laboratory facilities of King Saud University according to validated analytical protocols.

Urinary cotinine concentrations were quantified using LC-MS/MS due to its high analytical sensitivity and specificity for nicotine metabolite detection. Method validation included assessment of linearity, precision, accuracy, recovery, and reproducibility according to ICH-M10 bioanalytical method validation guidelines.

### LC-MS/MS analysis

Liquid chromatography high resolution mass spectrometry (LC-MS/MS) was carried out at King Abdullah International Medical Research Center (KAIMRC), Riyadh, Saudi Arabia. The cotinine stock solution was prepared by dissolving 50 mg of cotinine reference standard in 50 mL of UPW directly in its original vial, resulting in a final concentration of 5 mg/mL. From this primary stock, a secondary stock solution was prepared at a concentration of 1 mg/mL by dilution with UPW. The secondary stock was then aliquoted into labeled tubes and stored at –20 °C until use to avoid repeated freeze–thaw cycles. For the internal standard, 2-Phenylimidazole (2PI), a 0.1 M stock solution was prepared by accurately weighing 14.42 mg of 2PI (MW = 144.17 g/mol) and dissolving it in 1 mL of methanol. From the 1 mg/mL COT stock solution, working solutions of 1, 10, and 100 µg/mL were prepared. From the 0.1 M 2PI stock, a 10 mM working solution was prepared by diluting 100 µL of the 0.1 M stock with 900 µL of methanol in a 1 mL Eppendorf tube. All working solutions were stored at –20 °C until required for analysis.

### Urine sample preparation procedure

Following the sample preparation method described previously [25], extraction of the analytes from urine was performed using a simple protein precipitation technique with acetonitrile. A volume of 450 μL of acetonitrile containing IS was added to 150 μL of the urine sample. The mixture was then centrifuged at 15,000 rpm for 10 minutes, and 150 μL of the resulting supernatant was diluted with 600 μL of distilled water. The prepared solution was subsequently transferred directly to LC vials and then loaded into LC–MS/MS autosampler for analysis.

### Calibration curve samples

To prepare a set of urine-based cotinine calibration standards for LC-MS/MS analysis, a stock solution of cotinine at a concentration of 1 mg/mL (1,000,000 ng/mL) was used. The highest calibration standard required was 500 ng/mL, and this was used to generate lower concentration levels through serial dilution using blank urine as the matrix. Each calibration level was prepared in a final volume of 1,000 µL (1 mL), except the initial 500 ng/mL solution, which was prepared in a larger volume for use as a parent dilution. To prepare 10 mL of the 500 ng/mL cotinine-spiked urine, 5 µL of the 1 mg/mL cotinine stock solution was added to 9,995 µL of blank urine. This spiked sample was then vortexed thoroughly to ensure homogeneity. The resulting solution served as the highest calibration point and a parent solution for serial dilution of lower levels. Subsequent calibration levels (500, 375, 250, 100, 50, 25, 10, 5 and 2 ng/mL) were prepared by serial dilution of the 500 ng/mL solution using blank urine. The table below summarizes the volumes used for each dilution step.

### Quality control samples preparation

The QC samples prepared from the same matrix (urine), with three different levels (low, medium and high). The concentrations of the QCs were 15, 75, 375 ng/mL spiked urine, and the IS concentration is 30 ng/mL.

### Mobile phase preparation

The mobile phase was prepared following the method described previously [26]. It consisted of 0.1% ammonium hydroxide (NH₄OH) in water (v/v) as solution A (aqueous phase) and acetonitrile as solution B (organic solvent); however, in this study, a concentration of 0.01% NH₄OH was used instead.

### LC-MS/MS conditions

Chromatographic separation was performed using an Agilent 1260 binary pump LC system coupled with a SCIEX QTRAP 5500 mass spectrometer. The analytical column compartment was maintained at 20°C throughout the analysis. The mobile phase consisted of solvent A (aqueous solution) and solvent B (acetonitrile as organic solvent). Gradient elution was carried out with a constant flow rate of 0.1 mL/min as follows: The injection volume was set to 1 µL, and the autosampler temperature was maintained at 20°C. The sample draw speed was 200 µL/min, and the eject speed was also 200 µL/min. An integrated Valco diverter valve was utilized, configured to divert flow to waste (Position A) from 0 to 0.9 min, and directed to the MS detector (Position B) from 0.9 min to 10 min. The total run time per analysis was approximately 10 minutes. Detection was performed using multiple reaction monitoring (MRM) with positive polarity. The transitions, dwell times, and collision energies (CE) were set as follows: The LC-MS/MS system was operated using a TurboIonSpray source in positive ionization mode with the following parameters: Curtain Gas (CUR): 20 psi, Collision Gas (CAD): Medium, Ion Spray Voltage (IS): 4500 V, Source Temperature (TEM): 500 °C, Ion Source Gas 1 (GS1): 60 psi, Ion Source Gas 2 (GS2): 40 psi.

### Method validation

The analytical method used for cotinine quantification was validated and published earlier [26]. The validation procedures in this study followed the guidelines recommended by the International Council for Harmonization (ICH-M10) for bioanalytical method validation. Parameters assessed included calibration curve linearity, accuracy and precision. The calculations were done by Excel. The linearity of the method was evaluated by analysing nine calibration curves across different days. Each calibration curve included concentrations of 2, 5, 10, 25, 50, 100, 250, 375, and 500 ng/mL, and was prepared by spiking blank urine samples. The calibration range extended from the Lower Limit of Quantification (LLOQ), representing the lowest concentration that could be quantified with acceptable accuracy and precision, to the Upper Limit of Quantification (ULOQ), representing the highest concentration that could be quantified under the same criteria. The accuracy (recovery%) and precision (%CV) were assessed for the calculated concentrations of each calibrator. According to acceptance criteria, recovery was required to fall within ±15% of the nominal concentration, and %CV was not to exceed 15%, except for the LLOQ, where recovery was allowed within ±20% and %CV not to exceed 20%. The correlation coefficients (R²) of the calibration curves were also determined to confirm the linearity of the method.

### Accuracy and precision

The accuracy of the method was assessed by analyzing QC samples within a single analytical run (intraday) and across multiple analytical runs on different days (interday). The QC samples were prepared by spiking blank urine with different concentration levels of cotinine (15, 75, and 375 ng/mL) and the internal standard (2PI) at 30 ng/mL. The QC samples were analyzed after the calibration curve, and their measured concentrations were evaluated for accuracy by calculating the recovery percentage using the formula: (mean measured concentration / nominal concentration) × 100. This assessment was applied across all QC levels, including low (LQC), medium (MQC), and high (HQC) controls. Method precision, also referred to as method reproducibility, was evaluated by calculating the coefficient of variation (CV%) for each QC level using the formula: (standard deviation / mean concentration) × 100. Acceptance criteria required recovery% to be within ±15 of the nominal concentration, and the %CV not to exceed 15%.

### Data integration between questionnaire and biomarker results

Following LC-MS/MS analysis, urinary cotinine concentrations were matched with questionnaire responses using unique participant identification codes to generate an integrated dataset containing both biochemical and behavioural data.

### Cutoff determination

To determine an appropriate urinary cotinine cutoff value for distinguishing smokers from nonsmokers, a Receiver Operating Characteristic (ROC) curve analysis was conducted using GraphPad Prism software. The analysis aimed to optimize sensitivity and specificity in classifying participants based on their self-reported smoking status. To apply this, cotinine was used as the continuous variable, and smoking status was the binary variable.

### Statistical analysis

Statistical analysis was performed using GraphPad Prism (version 10.4.2). Both the t-test and analysis of variance (ANOVA) were applied to compare differences between groups. The data were plotted, and the software was used to conduct complete data analysis. Data distribution and homogeneity of variances were assessed prior to inferential analyses. Because urinary cotinine concentrations exhibited skewed distributions and significant heteroscedasticity, pairwise group comparisons were performed using Welch’s corrected unpaired t-test. Median and interquartile range (IQR) values were additionally reported to better characterize non-normally distributed data and reduce the influence of extreme observations. Statistical analyses were conducted using GraphPad Prism software, and p-values < 0.05 were considered statistically significant. For visualization on logarithmic-scale graphs, samples with non-detectable urinary cotinine concentrations (0 ng/mL) were assigned a small positive value equal to the lower detection limit to enable plotting on the logarithmic axis. This graphical adjustment was applied solely for visualization and was not used in any statistical analyses.

## Results

### Questionnaire survey result

The results obtained from the self-administered questionnaire, and the response of the participants are summarized in the next subsections.

### Demographic characteristics of the participants

**Fig 1:**
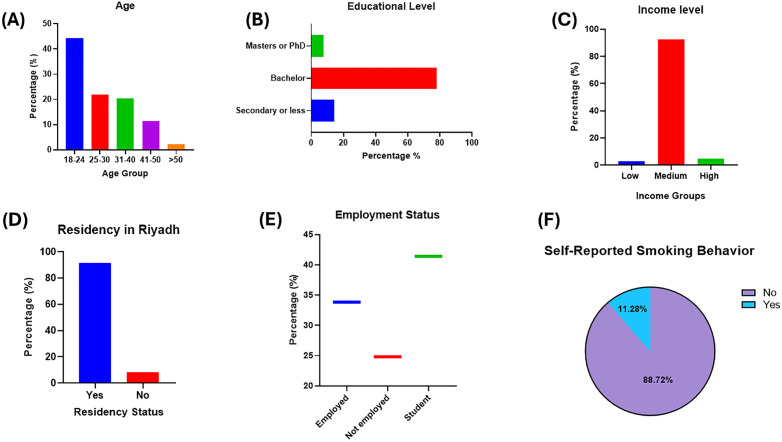
Percentages of Saudi female study participants categorized according to: A) age groups. B) Educational level, C) Income level, D) Residency status, E) Employment status, and F) self-reported smoking behaviour.

This analysis presents the responses from a cross-sectional survey conducted among 149 individuals to assess demographic characteristics and smoking behaviour. This number reflects the preliminary count before applying the exclusion criteria or verifying each urine sample with its corresponding questionnaire. After applying the eligibility criteria and ensuring complete matching, the final sample included 133 participants. The demographic characteristics of the study population were summarized in S1 Table. The analysis of the age distribution shows that the majority of participants were young adults. Specifically, 44.4% were between 18 and 24 years old, followed by 21.8% aged 25–30 years, and 20.3% aged 31–40 years. A smaller proportion of the sample fell into the older age groups, with 11.3% aged 41–50 years, and only 2.3% over the age of 50 (Fig 1A). With respect to education, the data reveals that the majority of participants were highly educated. Most subjects (78.2%) held a bachelor’s degree, while 14.3% had completed secondary education or less. A smaller proportion (7.5%) had attained postgraduate qualifications, such as a master’s degree or PhD (Fig 1B). In terms of income, the sample was predominantly grouped within the medium-income group. A total of 92.5% reported medium income, compared to only 3% in the low-income group and 4.5% in the high-income group (Fig 1C). Geographic residency data showed that 91.7% of the respondents were residents of Riyadh, with only 8.3% residing elsewhere (Fig 1D). Employment status was also explored. Among respondents, 41.4% identified as students, 33.8% were employed, and 24.8% were unemployed (Fig 1E). The participants were asked about their smoking status. The majority reported that they did not smoke (88.7%), while only 11.3% indicated that they were smokers (Fig 1F).

### Smoking status and history

**Fig 2:**
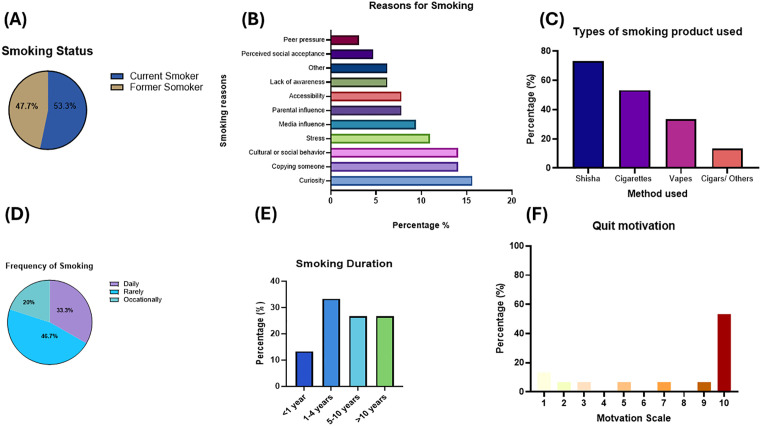
Percentages of Saudi female study participants categorized according to: A) Smoking status, B) Reasons of smoking, C) Smoking product types, D) Smoking frequency, E) Smoking duration, and F) Measurement of quit motivation scores.

The total smoker participants classified as either current or former smokers. Of these, 53.3% were current smokers, while 46.7% classified themselves as former smokers (Fig 2A).

### Reasons for smoking among Saudi female study participants

Among the reasons given for smoking, curiosity was the most frequent reason for smoking in our participants (15.62%). This was followed by copying someone and cultural or social behavior (14.06% for each). Then, stress (10.94%), while media influence accounted for 9.38%. Other factors included parental influence and accessibility (7.81%), lack of awareness and other reasons (6.25%), perceived social acceptance (4.69%), and peer pressure (3.12%) (Fig 2B).

#### Types of smoking products and smoking frequency and duration among Saudi females.

Most of Saudi female smoker participants used either shisha or cigarettes. Shisha was the most commonly used product (73.3%), followed by cigarettes (53.3%). Also, vapes were used (33.3%), while cigars or other tobacco products were less commonly used with only 13.3% (Fig 2C). Most of Saudi female smoker participants reported smoking rarely (46.7%) or occasionally (20%), while only 33.3% smoked daily (Fig 2D). Most of Saudi female smoker participants had been smoking for more than one year, about 33.3% of them had been smoking for 1–4 years, while 26.7% had smoked for 5–10 years and another 26.7% for more than 10 years. Only 13.3% had smoked for less than one year (Fig 2E).

### Smoking quit attempts and motivation to quit smoking among Saudi female participants

The smoker participants were asked about their efforts to quit smoking, most of them reported having tried to quit at some point 60%, indicating a significant level of awareness or desire for change among this group. However, when asked how many times they had attempted to quit, 53% of smokers responded. Half of them reported attempting to quit 1–4 times, while the remaining half had tried 5–10 times or more than 10 times. Despite this, only 6.7% of the participants reported using a specific quitting method such as nicotine replacement therapy (NRT). The participants were asked to rate their motivation to quit smoking on a scale of 1–10, with 10 being extremely motivated. More than half of participants rated their motivation at 10 (53.3%), indicating a strong desire to quit, However, the remaining participants reported lower motivation levels, with (6.7–13.3%) selecting scores between 1 and 3. Moderate motivation levels (scores between 4 and 9) were reported by 5–10% of participants (Fig 2F).

### The percentage of second-hand smoke among saudi female participants

To identify the second-hand smokers, the relationship between participants’ smoking status and whether they live with a smoker in the family were assessed (S2 Table in S1 File). Higher percentage was reported for Saudi women’s second-hand smokers who did not smoke but reported living with a smoker (45.9%). Additionally, a higher percentage (42.9%) was recorded for nonsmokers and those who lived in smoke-free homes. While less percentage was reported for Saudi women’s who smoke and also lived with another smoker (9.0%) or who smoke but did not live with any smoker (2.3%). Among the participants who reported second-hand smoke exposure but did not smoke themselves, follow-up data were collected to identify the family member responsible for the exposure. Each response was analysed to count all mentioned individuals. The most frequently reported source of second-hand smoke was the brother (mentioned 25 times), followed by the father (20 times), and the husband (13 times). Other reported sources included the mother (2 mentions), sister (1 mention), and other relatives (17 mentions).

### Perceived health effects of smoking

In terms of health outcomes, a majority of respondents (85.4%) stated that they had not experienced any health problems related to smoking. However, 14.6% did report smoking-related health issues. When asked to specify these issues, responses included respiratory conditions such as asthma, chronic cough, wheezing, and shortness of breath, as well as cardiovascular complaints. Notably, 12.2% of participants mentioned specific respiratory symptoms, and 4.9% reported chest pain or tightness. Participants who identified as smokers were also asked to share their reasons for quitting or attempting to quit smoking. Among the 15 respondents, the most commonly cited reasons were health concerns (33.3%) and long-term planning (26.7%). Additional reasons included improved physical fitness (20%), pregnancy (20%), and family or social relationships (20%). Financial concerns and personal appearance were each reported by 13.3% of respondents.

### Open-ended response

Most participants did not leave any additional comments. However, a few shared valuable insights and suggestions. Some participants emphasized the importance of using natural aids, such as herbs or *miswak*, to support smoking cessation. Others highlighted the need for strong public enforcement of smoking bans in non-smoking areas, expressing concern about the health risks posed by second-hand smoke. Several responses reflected frustration, particularly from nonsmokers exposed to smoke, who felt their health was more affected than smokers themselves.

A few participants suggested promoting walking, eating fruit, or adopting other healthy habits as alternatives whenever the urge to smoke arises. One response described smoking as a temporary escape from reality rather than a real solution to underlying problems. Another pointed out that individuals trying to quit often shift to seemingly less harmful methods, such as shisha or electronic smoking devices, although they remain unaware of the risks. Some participants recommended attending specialized clinics or addiction centers for support. Others expressed the need for more public campaigns to raise awareness and offer help. A few highlighted the role of prayer and personal motivation in quitting smoking.

### Analysis of urine cotinine levels

**Fig 3:**
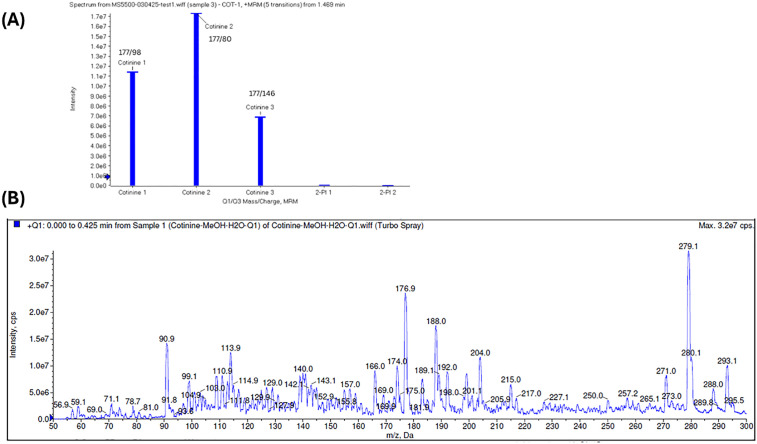
A) Extracted ion chromatograms of cotinine with transitions: 177/80 (quantifier), 177/98, and 177/146 (qualifiers). **B)** Precursor ion scan of the cotinine compound: The Q1 scan, which detects the precursor ion of cotinine at 176.9 m/z in a 50:50 MeOH:H₂O solution, showed an intensity of approximately 2.2 × 10^7^ cps.

Cotinine was quantified to assess tobacco smoke exposure levels among Saudi female study participants using LC-MS/MS. A total of 133 urine samples were analyzed using LC-MS/MS by monitoring one quantifier ion (177/80) and two qualifier ions (177/98 and 177/146). A sample was classified as detected if the quantifier transition was present along with at least one qualifier transition. The choice of quantifier ion was based on signal intensity among cotinine transitions. As shown in Fig 3A, the 177/80 transition exhibited the strongest signal and was therefore selected for quantification. Based on this detection criterion, 48 samples showed detectable cotinine concentrations, while 85 samples were classified as not detected. Among the detected samples, cotinine concentrations measured by the quantifier ion ranged from 0.02 ng/mL to 7.693 ng/mL, indicating a wide variability in nicotine exposure levels.

### Optimization LC and MS conditions

Direct infusion of COT and 2PI solutions into the mass spectrometer was performed to obtain precursor ion scans for both compounds. Subsequently, product ion scans were conducted for each compound. As shown in Fig 3B, the scan ranges from 50 to 300 m/z (Da) identified COT at 176.9 m/z with an intensity of 2.5 × 10⁷ cps. The COT was prepared in a 50:50 (v/v) methanol-water solution. As observed in S1 Fig in S1 File, Formic Acid (FA) shows the highest signal intensity, but Acetic Acid (AA) improves ionization slightly less effective than formic acid (FA). Ammonium Bicarbonate (AB) and Ammonium Formate (AF) exhibit similar ionization effects and signal intensities.

**Fig 4:**
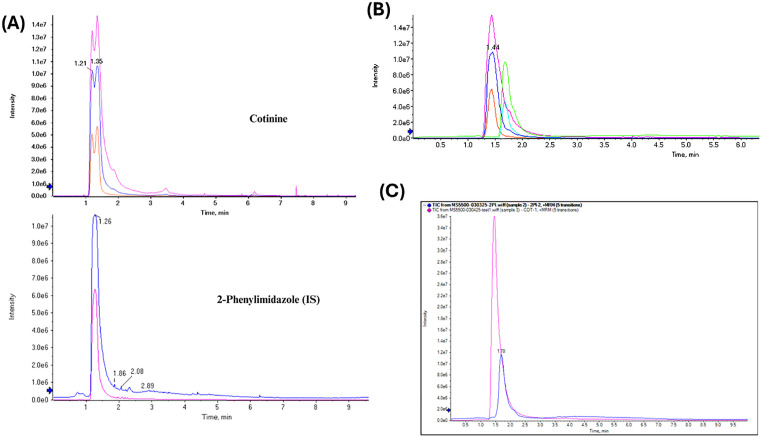
A) Chromatographic profiles of COT (top) and 2PI (bottom) dissolved in methanol. **B)** Chromatogram of COT with 2PI dissolved in water. **C)** Cotinine and 2-Phenylimidazole peak appearance with using 0.1% Ammonium hydroxide.

Following the initial MS tuning, a solvent optimization step was conducted to assess its impact on chromatographic peak shape and signal clarity. Two standard solutions were prepared containing 5 µM of COT and 2PI. Initially, the standards were dissolved in methanol (MeOH) and injected into the LC-MS system for peak evaluation. As seen in the chromatograms at Fig 4A, the analyte peaks were detected but exhibited a clipped appearance in COT and tailing in 2PI, indicating poor peak symmetry, likely caused by an injection solvent strength incompatible with the mobile phase. To address this, the injection solvent was changed from MeOH to H₂O. Upon reanalysis (Fig 4B), the chromatographic peaks significantly improved in shape with well-defined apexes and without clipping.

This indicates that water is a more suitable injection solvent for these analytes under the current chromatographic conditions, thereby improving peak shape. This finding underscores the importance of matching injection solvent strength with the starting conditions of the LC gradient.

The concentration of NH₄OH in both the A and B solutions was adjusted because COT and 2PI exhibited tailing, peak overlap, and increased noise (Fig 4C). Therefore, a concentration of 0.01% was tested, which resulted in improved peak shape and a better signal-to-noise ratio.

### Method validation

#### Calibration curve evaluation.

The performance of the calibration curve across nine concentration levels was summarized in S1 Fig in S1 File, ranging from 2 ng/mL to 500 ng/mL, based on seven replicate injections per level. The mean recovery values ranged from 90.13% to 107.16%, indicating acceptable analytical accuracy across the entire calibration range. The method’s precision, expressed as the coefficient of variation (%CV), was within the generally accepted limit of ≤15% for all levels, except at the lowest concentration (2 ng/mL), which remained within the ≤ 20% threshold recommended by ICH-M10 guidelines for the lower limit of quantification (S3 Table in S1 File).

Calculated concentrations were obtained from LC-MS/MS analysis using MultiQuant 3.0.3 and plotted in GraphPad Prism to evaluate linearity. As shown in S2 Fig in S1 File, each replicate is represented by a distinct symbol and colour, and the calculated concentrations are plotted against the nominal concentrations to assess linearity. The coefficient of determination (R²) values for the seven curves ranges from 0.9911 to 0.9995, indicating excellent linearity across all replicates.

#### Accuracy and precision evaluation.

The accuracy and precision of the analytical method for urinary cotinine quantification were evaluated according to the ICH-M10 guidelines. Both intraday (within-run) and interday (between-run) validations were performed at three QC levels: LQC (15 ng/mL), MQC (75 ng/mL) and HQC (375 ng/mL), using six replicate measurements per level. For intraday validation, the mean calculated concentrations were 13.04 ng/mL for LQC, 71.75 ng/mL for QC, and 412.55 ng/mL for HQC. The corresponding mean recoveries were 86.98%, 95.67%, and 110.01%, all within the acceptable range of 85–115%, indicating good analytical accuracy. The CV% was 9.21%, 4.55%, and 4.84% for LQC, QC, and HQC, respectively, confirming that intraday precision met the criteria of ≤15% CV%. In the interday validation, the method demonstrated consistent performance across different analytical runs. The recovery values which demonstrate accuracy were 90.35%, 99.23%, and 99.43%. Precision, as reflected by CV%, was 3.92% for LQC, 8.51% for QC, and 6.19% for HQC. Both intraday and interday validation results confirm that the method is accurate, precise, and reproducible, in compliance with ICH-M10 guidelines, and is therefore suitable for the quantitative determination of cotinine in urine samples across a broad concentration range (Table 1).

**Table 1:** The accuracy and precision validation at low, medium, and high QC levels.

Validation type	Intraday validation			Interday validation		
Nominal concentration (ng/mL)	LQC (15)	QC (75)	HQC (375)	LQC (15)	QC (75)	HQC (375)
**Calculated concentration (ng/mL)**	14.42	75.52	375.8646	13.09	64.22	373.76
	14.34	68.95	403.3529	13.48	75.51	331.07
	12.19	74.21	427.8966	13.96	72.88	372.60
	11.92	66.90	421.3584	14.41	73.06	398.62
	11.85	73.10	426.2487	13.08	77.41	388.64
	13.54	71.84	420.5867	13.30	83.48	372.57
**Mean**	13.04	71.75	412.5513	13.55	74.42	372.88
**Recovery (%)**	86.98	95.67	110.0137	90.35	99.23	99.43
**SD**	1.20	3.27	19.97876	0.53	6.33	23.07
**CV (%)**	9.21	4.55	4.842733	3.92	8.51	6.19

#### Correlation between cotinine levels and self-reported smoking status.

Cotinine levels were assessed mong Saudi female study participants and classified as nonsmokers, passive smokers, and smokers and were compared with the self-reported questionnaire data.

### Cotinine detection by smoking status

The Table 2 presents a summary of urinary cotinine levels across smokers, nonsmokers, and passive smokers. According to the self-reported questionnaire, a total of 15 smokers, 57 nonsmokers, and 61 passive smokers were included. Biomarker-based classification using the urinary cotinine cutoff was performed separately and is presented in the ROC and cutoff determination analyses. Smokers demonstrated the highest cotinine concentrations, with a mean value of 1511 ng/mL and a maximum reaching 7693 ng/mL. The standard deviation (2606 ng/mL) observed among smokers was high and associated with a wide range. In contrast, nonsmokers exhibited very low cotinine levels, with both the 25th percentile and the median at 0 ng/mL, and a mean of 2.31 ng/mL. However, passive smokers showed slightly elevated cotinine levels compared to nonsmokers, with a mean of 15.93 ng/mL and a maximum of 868.7 ng/mL. However, the median remained at 0 ng/mL, indicating that most passive smokers had only limited exposure. By comparing the mean cotinine levels across the different categories, smokers exhibited a markedly higher mean cotinine concentration compared to passive smokers and nonsmokers (S2 Fig in S1 File). Because urinary cotinine concentrations demonstrated marked skewness and unequal variances, median and interquartile range (IQR) values were additionally reported. Smokers showed substantially higher urinary cotinine concentrations [median (IQR): 66.99 (7.85–4491) ng/mL] compared with nonsmokers [0.00 (0.00–0.86) ng/mL] and passive smokers [0.00 (0.00–1.27) ng/mL].

**Table 2:** Descriptive statistics of urinary cotinine concentrations (ng/mL) among smokers, nonsmokers, and passive smokers, including median and interquartile range (IQR) values.

	Smoker cotinine level	Nonsmoker cotinine level	Passive smoker cotinine level
Number of values	15	57	61

Minimum	0.000	0.000	0.000
25% Percentile	7.852	0.000	0.000
Median	66.99	0.000	0.000
75% Percentile	4491	0.8617	1.272
Maximum	7693	46.28	868.7
Range	7693	46.28	868.7

Mean	1511	2.305	15.93
Std. Deviation	2606	7.886	111.1
Std. Error of Mean	673.0	1.045	14.23
Median (IQR)	66.99 (7.85-4491)	0.00 (0.00-0.86)	0.00 (0.00-1.27)

### Statistical analysis

#### One-Way ANOVA.

The analysis revealed a statistically significant difference among group means (p < 0.0001), with an R² value of 0.2384, indicating that approximately 23% of the variability in cotinine levels could be explained by smoking status. Both the Brown-Forsythe and Bartlett’s tests were performed to assess the assumption of homogeneity of variances. Results from the Brown-Forsythe test (F (2, 130) = 20.06, p < 0.0001) and the Bartlett’s test (Bartlett’s statistic = 727.7, p < 0.0001) confirmed that there were significant differences in variances between the groups (S4 Table in S1 File).

### Pairwise comparisons using unpaired t-Tests

#### Smoker vs Nonsmoker comparison.

The unpaired t-test was performed to compare urinary cotinine levels between smokers and nonsmokers (Table 3). Urinary cotinine concentrations demonstrated marked skewness and unequal variances across study groups. Therefore, Welch’s corrected unpaired t-test was applied for pairwise comparisons, and median with interquartile range (IQR) values were additionally reported to better represent data distribution. The analysis revealed a statistically significant difference between the two groups, indicating that smokers had significantly higher cotinine levels compared to nonsmokers. The mean cotinine concentration for smokers was 1511 ng/mL, whereas for nonsmokers it was 2.30 ng/mL, resulting in a mean difference of −1509 ± 338.3 ng/mL. The 95% confidence interval for the difference between means ranged from 2183 to 834.0 ng/mL. The calculated R² was 0.2213, suggesting that approximately 22% of the variation in cotinine levels could be explained by smoking status. In addition, an F-test demonstrated a significant difference in variance between the two groups (F = 109241, p < 0.0001), showing that the variances between the groups were not equal. Median urinary cotinine concentrations were substantially lower than mean values across groups, confirming skewed distributions and the influence of extreme observations. Smokers exhibited substantially higher urinary cotinine concentrations compared with both nonsmokers and passive smokers. Median (IQR) cotinine concentrations were 66.99 (7.85–4491) ng/mL in smokers, 0.00 (0.00–0.86) ng/mL in nonsmokers, and 0.00 (0.00–1.27) ng/mL in passive smokers. Welch’s corrected unpaired t-test demonstrated statistically significant differences between smokers and nonsmokers (p < 0.0001) as well as between smokers and passive smokers (p < 0.0001), whereas no significant difference was observed between passive smokers and nonsmokers (p = 0.3576).

**Table 3:** Pairwise comparison of urinary cotinine concentrations between study groups using Welch’s corrected unpaired t-test.

Groups Compared	Smoker vs Nonsmoker	Smoker vs Passive Smoker	Passive Smoker vs Nonsmoker
Test Used	Welch’s corrected unpaired t-test (Two-tailed)	Welch’s corrected unpaired t-test (Two-tailed)	Welch’s corrected unpaired t-test (Two-tailed)
Sample Size (Smoker)	15	15	57
Sample Size (Nonsmoker)	57	61	61
Mean (Smoker)	1511 ng/mL	1511 ng/mL	2.305 ng/mL
Mean (Nonsmoker)	2.305 ng/mL	15.93 ng/mL	15.93 ng/mL
Median concentration (ng/mL)	66.99 vs 0.00	66.99 vs 0.00	0.00 vs 0.00
Interquartile range (IQR)	7.85-4491 vs 0.00-–0.86	7.85-4491 vs 0.00–1.27	0.00-1.27 vs 0.00–0.86
Mean difference ± SE* (ng/mL)	−1509 ± 338.3 ng/mL	−1495 ± 328 ng/mL	13.63 ± 14.76 ng/mL
95% Confidence Interval	−2183 to −834.0 ng/mL	−2149 to −841.4 ng/mL	−15.60 to 42.86 ng/mL
t-value, df**	2.325	2.280	0.948
P-value	< 0.0001	< 0.0001	0.3576
Statistical significance	Significant	Significant	Not significant
F-test for Variances (F, DFn, DFd)	109241 (14, 56)	550.2 (14, 60)	198.6 (60, 56)
Variance equality assessment (F-test)	p < 0.0001	p < 0.0001	p < 0.0001
Variance interpretation	Unequal variances	Unequal variances	Unequal variances

Pairwise comparisons were performed using Welch’s corrected unpaired two-tailed t-test because homogeneity of variance was rejected by F-tests (P < 0.0001). Median and interquartile range (IQR) are additionally presented because urinary cotinine concentrations were highly skewed.*SE, standard error

** df, degree of freedom

**Fig 5:**
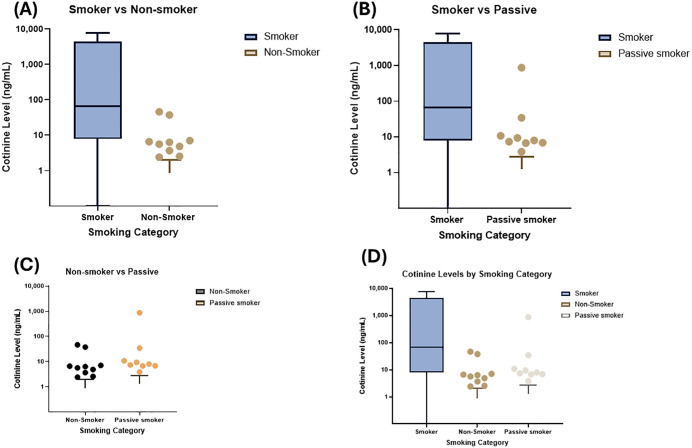
A) Boxplot showing urinary cotinine concentrations (ng/mL) among smokers and nonsmokers, displayed on a log scale 1- 10000 ng/mL. **B)** Boxplot showing urinary cotinine concentrations (ng/mL) among smokers and passive smokers, displayed on a log scale 1- 10000 ng/mL. **C)** Box-plot showing urinary cotinine concentrations (ng/mL) among nonsmokers and passive smokers, displayed on a log scale 1- 10000 ng/mL. **D)** Boxplot showing urinary cotinine concentrations (ng/mL) among smokers, passive smokers, and nonsmokers on a logarithmic scale. Zero (non-detectable) urinary cotinine concentrations were plotted using the lower detection limit solely for logarithmic visualization.

The box plot (Fig 5A) illustrates the distribution of urinary cotinine concentrations among smokers and nonsmokers, displayed on a logarithmic scale ranging from 1 to 10,000 ng/mL. Smokers exhibited significantly higher cotinine levels compared to nonsmokers. The interquartile range was broad, and the median value was markedly elevated relative to the nonsmoker group, reflecting considerable variability within the smoker population. In contrast, nonsmokers demonstrated cotinine concentrations that were tightly clustered at the lower end of the scale, with individual values ranging between 1 and 50 ng/mL. Overall, the figure visually underscores the significant difference in urinary cotinine levels between smokers and nonsmokers.

#### Smoker vs Passive smoker comparison.

The unpaired two-tailed t-test was performed to compare cotinine levels in urine between smokers and passive smokers. Smokers had a significantly higher mean cotinine concentration (1511 ng/mL) compared to passive smokers (15.93 ng/mL), with a mean difference of –1495 ± 328.0 ng/mL (t(74) = 4.558, p < 0.0001). The 95% confidence interval for the difference ranged from –2149 to –841.4 ng/mL. The effect size (R²) was 0.2192, indicating that approximately 22% of the variation in cotinine levels could be attributed to smoking status. An F-test for variances revealed a significant difference (F(14, 60) = 550.2, p < 0.0001), suggesting heterogeneity of variances between the two groups. This boxplot in Fig 5B shows urinary cotinine concentrations among smokers and passive smokers on a logarithmic scale. Smokers exhibited substantially higher and more variable cotinine levels, while passive smokers demonstrated lower concentrations with a narrower distribution. Individual data points are plotted to display the spread within each group.

#### Passive smoker vs Nonsmoker comparison.

An unpaired two-tailed t-test was performed to assess differences in urinary cotinine levels between passive smokers and nonsmokers. Although the mean cotinine concentration was higher among passive smokers (15.93 ng/mL) compared to nonsmokers (2.30 ng/mL), the difference was not statistically significant (t (116) = 0.9236, p = 0.3576). The mean difference was 13.63 ± 14.76 ng/mL, with a 95% confidence interval ranging from –15.60 to 42.86 ng/mL. The calculated effect size (R²) was 0.0073, indicating that exposure category explained only a minimal proportion of the variance in cotinine levels. An F-test for equality of variances showed a significant difference between the groups (F (60, 56) = 198.6, p < 0.0001), indicating that variances were unequal. The plot in Fig 5C displays urinary cotinine concentrations among passive smokers and nonsmokers. Both groups showed low cotinine levels, mostly clustered between 1 and 100 ng/mL. Although passive smokers had slightly higher median concentrations than nonsmokers, the overlap between the groups was considerable. Individual data points are shown to illustrate the spread, with one notable outlier observed among passive smokers.

Overall, the box-and-whisker plot (Fig 5D) presents urinary cotinine levels among smokers, passive smokers, and nonsmokers, displayed on a logarithmic Y-axis ranging from 1 to 10,000 ng/mL. Among smokers, cotinine concentrations exhibited the highest levels and the greatest variability, ranging from values near 1 ng/mL up to approximately 10,000 ng/mL. The box represents the interquartile range (25th to 75th percentile), while the horizontal line inside the box marks the median concentration. The spread among smokers was substantial, with several outliers observed beyond the whiskers.

In contrast, passive smokers and nonsmokers showed much lower cotinine concentrations, tightly clustered around the lower end of the scale. Both groups demonstrated relatively narrow distributions, with cotinine levels primarily concentrated between 5 and 100 ng/mL. Although a few individual outliers were present, the overall variability was considerably less compared to the smoker group.

### Refinement of smoking classification based on cotinine levels and self-reported behavior

As shown in the smoking participant category, three individuals exhibited undetectable cotinine levels (i.e., 0 ng/mL), which were interpreted as “not detected.” Although these participants had responded “yes” to the question “Do you smoke?”, further analysis of their questionnaire responses and smoking behaviour revealed that they were, in fact, former smokers. Based on this finding, we refined the smoking classification to support accurate determination of the cutoff value. The S5 Table in S1 File presents the classification of the 15 self-reported “smokers” along with their corresponding sample IDs: Ex-smokers were reclassified based on their response to the question, “Do you have a smoker in your family?” If the response was “yes,” they were categorized as passive smokers. If the response was “no,” they were reclassified as nonsmokers. Following this refinement (S6 Table in S1 File), the updated distribution of smoking status among participants is as follows: 11 were classified as smokers, 62 as passive smokers, and 60 as nonsmokers.

### Cutoff determination result

Urinary cotinine concentrations, quantified using the validated LC-MS/MS method, were analyzed as a continuous variable, while self-reported smoking status (smoker vs. nonsmoker) was used as the reference binary variable. Receiver operating characteristic (ROC) curve analysis was performed to determine the optimal cutoff value for distinguishing smokers from nonsmokers (Table 4). The ROC curve plotted sensitivity (true positive rate) against specificity (false positive rate) across a wide range of cotinine thresholds. Sensitivity remained at 100% for cutoff values between 0.036 and 14 ng/mL, indicating that all self-reported smokers were correctly identified within this interval. In contrast, specificity progressively increased with higher cutoff concentrations. Specificity reached 85% at 2.5 ng/mL, 90% at 5.3 ng/mL, 92% at 6.0 ng/mL, 95% at 6.8 ng/mL, and 97% at 14 ng/mL. Although a threshold of 14 ng/mL provided the highest specificity while maintaining 100% sensitivity, cutoff values above 6.0 ng/mL already demonstrated strong discriminatory performance with high specificity and favorable likelihood ratios. Considering both statistical performance and practical applicability for population-based classification, a cutoff value of 7.0 ng/mL was selected as a balanced and robust threshold for distinguishing smokers from nonsmokers in this study population. The ROC curve (S3 Fig in S1 File) demonstrates good preliminary discriminatory performance, with a sharp rise toward the upper-left corner and clear separation from the diagonal reference line, indicating high overall classification accuracy. ROC analysis demonstrated good discriminatory performance of urinary cotinine for distinguishing smokers from nonsmokers, with an area under the curve (AUC) of 0.94 (95% CI: 0.88–0.99, p < 0.001). The inclusion of ex-smokers and individuals with passive exposure within the self-reported smoker reference group may have introduced heterogeneity, potentially affecting the stability and precision of the ROC-derived cutoff value. Additionally, overlap between heavy passive exposure and light active smoking may have led to potential misclassification when relying solely on urinary cotinine concentrations. Accordingly, the proposed urinary cotinine cutoff value should be interpreted as preliminary and requires external validation in larger and more representative populations before broader application.

**Table 4:** Sensitivity, specificity, and likelihood ratios for various urinary cotinine concentration thresholds used to distinguish smokers from nonsmokers, as analysed using GraphPad Prism.

	Sensitivity%	95% CI	Specificity%	95% CI	Likelihood ratio
> 0.036	100	74% to 100%	65	52% to 76%	2.9
> 0.096	100	74% to 100%	67	54% to 77%	3.0
> 0.18	100	74% to 100%	68	56% to 79%	3.2
> 0.27	100	74% to 100%	70	57% to 80%	3.3
> 0.32	100	74% to 100%	72	59% to 81%	3.5
> 0.44	100	74% to 100%	73	61% to 83%	3.8
> 0.63	100	74% to 100%	75	63% to 84%	4.0
> 0.86	100	74% to 100%	77	65% to 86%	4.3
> 1.1	100	74% to 100%	78	66% to 87%	4.6
> 1.4	100	74% to 100%	80	68% to 88%	5.0
> 1.8	100	74% to 100%	82	70% to 89%	5.5
> 2.2	100	74% to 100%	83	72% to 91%	6.0
> 2.5	100	74% to 100%	85	74% to 92%	6.7
> 3.1	100	74% to 100%	87	76% to 93%	7.5
> 4.3	100	74% to 100%	88	78% to 94%	8.6
> 5.3	100	74% to 100%	90	80% to 95%	10
> 6.0	100	74% to 100%	92	82% to 96%	12
> 6.5	100	74% to 100%	93	84% to 97%	15
> 6.8	100	74% to 100%	95	86% to 99%	20
> 14	100	74% to 100%	97	89% to 99%	30
> 30	91	62% to 100%	97	89% to 99%	27
> 42	91	62% to 100%	98	91% to 100%	55
> 54	91	62% to 100%	100	94% to 100%	

### Smoking status based on the determined cutoff

Based on cotinine concentration analysis, a cutoff value of 7 ng/mL was used to classify individuals as smokers. Using this threshold, 16% of the participants were identified as smokers, while the remaining 84% were classified as non-smokers. This classification was based only on the biochemical detection of cotinine in urine, regardless of self-reported smoking status.

## Discussion

This study evaluated tobacco smoke exposure among Saudi females using urinary cotinine as an objective biomarker in combination with self-reported questionnaire data. In addition, a validated LC-MS/MS method was applied for urinary cotinine quantification, and a preliminary urinary cotinine cutoff value was explored for distinguishing smokers from nonsmokers within the study population. Integrating biochemical verification with self-reported smoking behaviour provided a broader understanding of active and passive tobacco exposure patterns and highlighted the limitations of relying solely on questionnaire-based assessments [27]. The LC-MS/MS method demonstrated strong analytical performance (R^2^ ≥ 0.9992; recovery 90–107%; CV ≤ 10%), ensuring reliable quantification of cotinine and precise differentiation between smokers, nonsmokers, and passive smokers. Unlike surveys alone, biomarker analysis revealed hidden nicotine exposure patterns, highlighting the value of dual-method approaches for strengthening public health surveillance and guiding targeted cessation and prevention strategies [28,29].

A major finding of this study was the discrepancy between self-reported smoking status and biomarker-confirmed nicotine exposure. Although only 11.3% of participants identified themselves as smokers, urinary cotinine analysis revealed detectable nicotine exposure in a considerably larger proportion of participants. Similar discrepancies between self-reported smoking and biochemical verification have been reported previously among female populations in conservative cultural settings [30]. Social stigma and cultural sensitivities surrounding female smoking in Saudi Arabia may contribute to underreporting of smoking behaviors. During participant recruitment and questionnaire administration, several observations further supported the presence of social influence on reporting behaviour, including instances in which family members attempted to influence participant responses or participants appeared hesitant to discuss smoking openly.[31]. The study population consisted largely of university students, healthcare employees, and hospital visitors recruited through convenience sampling. Consequently, the findings should be interpreted as exploratory and may not fully represent the broader Saudi female population. Nevertheless, inclusion of participants from multiple educational and occupational backgrounds provided insight into diverse smoking exposure patterns among Saudi females. Future multicenter studies involving larger and more representative cohorts from different geographic and socioeconomic backgrounds are needed to externally validate these findings and improve generalizability. Smoking initiation among participants was primarily associated with curiosity, social influence, and imitation, while shisha smoking was commonly reported among smokers [32]. These findings are consistent with previous regional studies indicating that waterpipe smoking is often perceived as less harmful than cigarette smoking despite substantial nicotine exposure [33]. Such misconceptions highlight the importance of culturally tailored tobacco awareness campaigns and targeted smoking cessation programs [34].

The study also identified considerable second-hand smoke exposure among nonsmoking participants, affecting nearly half of nonsmokers, primarily from male household members such as brothers, fathers, and husbands [35]. This exposure represents a critical public health concern, especially considering that 1.3 million deaths globally are attributed to second-hand smoke each year [36]. Public health interventions and educational initiatives targeting male family members could reduce this risk effectively. Participant perceptions regarding smoking-related health risks likely influence their motivation to quit and the reporting accuracy of smoking-related health issues. Clearer communication and educational programs emphasizing tobacco-related health consequences could enhance quitting motivation and accuracy of self-reported data.

Qualitative feedback at the end of the survey provided additional insights into motivations and barriers to quitting; future studies could benefit from thematic categorization of such feedback to better design tailored interventions.

Urinary cotinine analysis effectively validated smoking status and exposure patterns [21]. Several cases illustrated the depth of nicotine dependence, including smokers with cotinine levels exceeding 4000 ng/mL, who also reported strong motivation to quit but limited use of cessation aids. Conversely, some self-identified smokers showed undetectable cotinine, later revealed to be ex-smokers misclassified by the questionnaire. An unexpected finding was that 20% of participants classified as smokers exhibited undetectable cotinine levels. Further examination revealed these individuals to be former smokers who had misunderstood the classification criteria at the outset of the survey, reporting rare smoking frequency. This suggests a need to refine the questionnaire design, clearly differentiating between current smokers and ex-smokers for example, by specifically asking ex-smokers when they last smoked. Conversely, 36% of participants overall exhibited detectable urinary cotinine, indicating both active and unrecognized passive smoke exposure. Nearly half of self-reported nonsmokers reported living with smokers primarily fathers and brothers resulting in a high rate of passive exposure (46.6%), significantly exceeding the broader Saudi estimate (13.7%) [30]. This discrepancy strongly indicates underreporting or misunderstanding in self-reported smoking behaviours and highlights household smoking as a significant source of involuntary nicotine exposure.

Further emphasizing this point, some nonsmokers exhibited cotinine levels above the established cutoff of 7 ng/mL. This finding likely reflects broader environmental exposure, suggesting limitations in the original survey question (“Do you have a smoker in your family?”). To more accurately capture passive exposure sources, future questionnaires should instead inquire broadly about smoking exposure in the participants’ surroundings, including friends, classmates, or colleagues. Notably, one self-identified nonsmoker had a significantly elevated cotinine concentration (868.7 ng/mL). This participant, a university student, reported frequent exposure to second-hand smoke from her father and brother, coupled with existing asthma. Her case clearly illustrates the critical health risks associated with passive smoking and underscores the need for targeted public health efforts aimed at reducing domestic second-hand smoke exposure, especially among individuals with respiratory vulnerabilities.

In establishing an appropriate cutoff, challenges arose due to significant variation in reported cotinine thresholds across different biological matrices and populations [37]. Previously published urinary cotinine cutoffs ranged widely from 31.5 ng/mL to 146 ng/mL [38–40], reflecting variations influenced by factors such as ethnicity, gender, and genetics [27]. In the current study, ROC curve analysis was used to determine an optimal cutoff of 7 ng/mL, achieving good preliminary discriminatory performance (sensitivity 100%, specificity 95%), thereby effectively distinguishing smokers from nonsmokers among Saudi females. Detailed survey analyses identified critical smoking patterns, motivations, and associated behaviours among study participants, further highlighting likely underreporting of smoking habits. Several self-identified nonsmokers or ex-smokers exhibited cotinine concentrations above the validated cutoff, indicating either active smoking or substantial passive exposure. Observational data collected during the survey reinforced the hypothesis of response bias driven by social desirability, cultural constraints, and stigma associated with female smoking, contributing significantly to discrepancies between self-reported and biochemical data. Thus, integrating objective biochemical markers alongside self-reports is crucial for accurately determining smoking prevalence and exposure patterns, ultimately enabling more effective public health strategies to reduce tobacco use among Saudi women.

### Limitations

Several limitations should be considered when interpreting the findings of this study. Recruitment challenges related to cultural sensitivities surrounding female smoking reduced the final sample size and limited the number of self-reported smokers, thereby restricting statistical power and external generalizability. In addition, the convenience sampling design indicates that the findings should be interpreted as preliminary and exploratory rather than representative of the broader Saudi female population. The questionnaire was specifically developed for this study and reviewed for clarity; however, formal psychometric validation was not performed. Urinary cotinine concentrations also demonstrated marked skewness, unequal variances, and extreme values, which may influence mean-based comparisons despite the application of complementary variance-robust statistical approaches. Furthermore, because urinary cotinine reflects nicotine exposure from all nicotine-containing products, inclusion of participants who reported vaping may have introduced exposure heterogeneity that could not be fully differentiated from combustible tobacco exposure. Accordingly, the proposed cutoff value should be considered preliminary and require external validation in larger and more representative cohorts. To minimize the impact of these limitations, the study employed a validated LC-MS/MS analytical method, standardized sample preparation procedures, biochemical verification alongside self-reported data, and variance-robust statistical analyses. Future studies should incorporate larger multicenter cohorts, more representative sampling strategies, creatinine-normalized urinary cotinine measurements, and expanded assessment of alternative nicotine biomarkers. Longitudinal investigations examining smoking behaviours, passive exposure, and cessation outcomes among Saudi women would further strengthen tobacco exposure surveillance and support the development of targeted public health interventions.

## Conclusion

This study demonstrates that urinary cotinine is a reliable and objective biomarker for assessing tobacco smoke exposure among Saudi females. The validated LC-MS/MS method enabled accurate quantification of cotinine and explored a preliminary urinary cotinine cutoff value of 7 ng/mL for differentiating smokers from nonsmokers within the study population. Integrating biochemical verification with self-reported questionnaire data revealed evidence of smoking underreporting, likely influenced by cultural and social factors, and confirmed substantial household second-hand smoke exposure. These findings highlight the importance of incorporating biomarker-based verification into epidemiological studies to improve exposure classification, reduce reporting bias, and strengthen tobacco control strategies among women in Saudi Arabia. Despite the limitations, the combined use of biochemical and survey-based approaches provided valuable insight into tobacco exposure patterns among Saudi women. Future studies should include larger and more geographically diverse populations, apply robust statistical and biomarker-based methodologies, and integrate cotinine testing into national tobacco surveillance programs. Additional investigation of alternative biomarkers and factors influencing cotinine metabolism may further refine tobacco exposure assessment. Public health efforts focusing on household smoking reduction, family-based smoking cessation programs, and smoke-free home policies may also contribute to reducing passive tobacco smoke exposure in this population.

## Supporting information

S1 File Supporting information file. (DOCX)
