# Supplementary material for: Biomarker-based assessment of tobacco smoke exposure in Saudi females via analysis of urinary cotinine level
Source: PLoS One. 2026 Aug 11;21(8):e0355166. doi: 10.1371/journal.pone.0355166 (PMC13460576; doi:10.1371/journal.pone.0355166)
Supplement: Supplementary file 1 [file pone.0355166.s001.docx]

**Supporting Information**

**Biomarker-based Assessment of Tobacco Smoke Exposure in Saudi Females via analysis of Urinary Cotinine level**

| **S‎1 Table.** Demographic Data of the Saudi female study participants (n=133). | | | |
| --- | --- | --- | --- |
| **Characteristic** | | **n** | **%** |
| **Age** | 18-24 | 59 | 44.36 |
|  | 25-30 | 29 | 21.80 |
|  | 31-40 | 27 | 20.30 |
|  | 41-50 | 15 | 11.28 |
|  | >50 | 3 | 2.26 |
| **Education level** | Secondary or less | 19 | 14.29 |
|  | Bachelor | 104 | 78.20 |
|  | Masters or PhD | 10 | 7.52 |
| **Riyadh Residency** | Yes | 122 | 91.73 |
|  | No | 11 | 8.27 |
| **Income Level** | Low | 4 | 3.01 |
|  | Medium | 123 | 92.48 |
|  | High | 6 | 4.51 |
| **Employment Status** | Employed | 45 | 33.83 |
|  | Not-employed | 33 | 24.81 |
|  | Student | 55 | 41.35 |

| **S2** **Table.** Percentage of smoking status by presence of a smoker in the family | | |
| --- | --- | --- |
| **Smoker in the Family** | **Participant Smokes** | **N (%)** |
| Yes | Yes | 12 (9.0) |
| Yes | No | 61 (45.9) |
| No | Yes | 3 (2.3%) |
| No | No | 57 (42.9%) |

| **S3** **Table.** Calibration Curve Concentrations vs Calculated concentration across seven replicate runs. | | | | | | | | | |
| --- | --- | --- | --- | --- | --- | --- | --- | --- | --- |
|  | **Nominal concentration (ng/mL)** | | | | | | | | |
|  | **2** | **5** | **10** | **25** | **50** | **100** | **250** | **375** | **500** |
| **Calculated concentration (ng/mL)** | 1.97 | 4.86 | 10.43 | 29.13 | 46.35 | 95.10 | 238.88 | 318.33 | 428.63 |
|  | 1.19 | 4.79 | 9.48 | 23.64 | 54.92 | 103.41 | 257.31 | 367.73 | 513.78 |
|  | 1.61 | 4.86 | 10.75 | 24.85 | 47.31 | 106.48 | 235.70 | 347.04 | 475.14 |
|  | 2.02 | 4.75 | 9.09 | 25.51 | 47.52 | 113.22 | 254.43 | 374.37 | 526.16 |
|  | 1.69 | 4.35 | 10.13 | 28.13 | 57.38 | 107.89 | 263.71 | 378.88 | 518.41 |
|  | 1.95 | 4.73 | 10.38 | 27.68 | 56.57 | 117.37 | 251.61 | 404.26 | 480.84 |
|  | 2.19 | 4.68 | 9.47 | 27.41 | 54.31 | 106.66 | 257.10 | 426.43 | 484.02 |
| **Mean** | 1.80 | 4.72 | 9.96 | 26.62 | 52.05 | 107.16 | 251.25 | 373.86 | 489.57 |
| **Recovery (%)** | 90.13 | 94.39 | 99.61 | 106.49 | 104.10 | 107.16 | 100.50 | 99.70 | 97.91 |
| **SD** | 0.34 | 0.18 | 0.62 | 1.98 | 4.79 | 7.09 | 10.26 | 35.48 | 33.63 |
| **CV (%)** | 18.59 | 3.72 | 6.19 | 7.44 | 9.20 | 6.62 | 4.08 | 9.49 | 6.87 |

| **S4** **Table.** Summary of one-way ANOVA and variance tests for urinary cotinine levels among smoking categories. *Note: there is a highly significant difference in variance across all tests applied.* | |
| --- | --- |
| **Analysis** | **Statistic** |
| **Data sets analyzed** |  |
| **ANOVA Summary** | F-value 20.35  P-value < 0.0001  R squared 0.2384 |
| **Brown-Forsythe Test** | F (DFn, DFd) 20.06 (2, 130)  P-value < 0.0001 |
| **Bartlett’s Test** | Corrected Bartlett’s statistic 727.7  P-value < 0.0001 |

| **S5 Table.** The classification of the 15 self-reported “smokers” along with their corresponding sample IDs | | |
| --- | --- | --- |
| **Smoker Classification** | **Count** | **Sample ID** |
| Current smokers | 8 | N40, 0509-05, N90, N91, N93, N95, N96, N97 |
| Ex-smokers | 7 | N6, N11, N35, N67, N68, 8, N94 |

| **S6 Table.** The breakdown of cotinine level in 15 self-reported “smokers” and “Ex-smokers”. | | |
| --- | --- | --- |
| **ID** | **Cotinine Level (ng/mL)** | **Notes** |
| N6 | Not-detected | Possible true ex-smoker |
| N11 | 7693.26 | High level—likely still smokes |
| N35 | Not-detected | Possible true ex-smoker |
| N67 | 5265.90 | High level—likely still smokes |
| N68 | 7.85 | Passive exposure (per questionnaire) |
| 8 | Not-detected | Possible true ex-smoker |
| N94 | 70.53 | Likely still smokes |

S1 Fig. Five Q1 mass spectra of cotinine obtained using direct infusion into MS under different solvent and additive conditions. Spectrum 1 (Top Panel): Cotinine in 50:50 H₂O/MeOH (No additive). Spectrum 2: Cotinine in 50:50 H_2_O/MeOH + 0.1% Formic Acid. Spectrum 3: Cotinine in 50:50 H_2_O/MeOH + 0.1% Acetic Acid. Spectrum 4: Cotinine in 50:50 H_2_O/MeOH + 5 mM Ammonium Bicarbonate. Spectrum 5 (Bottom Panel): Cotinine in 50:50 H_2_O/MeOH + 5 mM Ammonium Formate.

**S2 Fig**. The linear relationship between calibration curve of cotinine and calculated concentration across the range of 2–500 ng/mL. Each curve represents one analytical run, and the R² values (ranging from 0.9948 to 0.9995) indicate excellent linearity. Mean urinary cotinine concentrations across smoking categories.

**S3 Fig**. Receiver Operating Characteristic (ROC) curve for distinguishing smokers from nonsmokers based on urinary cotinine levels.
