# Supplementary material for: Biomarker-based assessment of tobacco smoke exposure in Saudi females via analysis of urinary cotinine level
Source: PLoS One. 2026 Aug 11;21(8):e0355166. doi: 10.1371/journal.pone.0355166 (PMC13460576; doi:10.1371/journal.pone.0355166)
Supplement: Supplementary file 2 [file pone.0355166.s003.docx]

**Author Reply List**

To

**Prof. Thiago P. Fernandes, PhD**

**Academic Editor**

**PLOS One**

First thanks for handling our manuscript and considering it for publication in PLOS ONE after careful revision. Kindly, find all our responses to the raised queries. The manuscript has been carefully revised.

 Review Comments to the Author

Reviewer #1: This manuscript addresses an important public health issue by combining biomarker-based assessment with self-reported data to evaluate tobacco exposure among Saudi females. While the study has merit, several methodological, statistical, and reporting issues need to be addressed before the conclusions can be adequately supported.

**Reply:** **We sincerely thank the reviewer for his thorough evaluation and constructive comments. We appreciate all the efforts made in reviewing our manuscript. We have carefully revised the manuscript to address all raised concerns.**

1. The manuscript contains multiple citation-formatting errors, including inconsistent use of superscripts (e.g., Lines 61, 62, 131, 133, 191, 215, and 220). Several in-text citations do not correspond to the reference list. Reference formatting should be corrected throughout to comply with journal guidelines.

**Reply:** **All in-text citations have been carefully revised to comply with journal formatting guidelines.**

2. The statement regarding underreporting of smoking among Saudi females due to cultural and social stigma (Lines 69-72) is not adequately supported by the cited references. Reference 5 reports prevalence among medical students but does not address stigma or underreporting, while Reference 6 discusses self-report bias in general populations without Saudi- or female-specific evidence. Consider citing <https://smj.org.sa/content/40/9/867>

**Reply:** **We have revised the relevant paragraph and appropriate references were added, including the suggested article from the Saudi Medical Journal.**
3. References 8 and 9 appear to cover overlapping content and should be reviewed for redundancy.

**Reply: We reviewed both references and removed redundancy. One citation was deleted.**
4. The Methods section states that a minimum sample size of 385 participants was calculated and that recruitment continued until this target was reached. However, the final analyzed sample comprises only 133 participants. This discrepancy is not explained and raises concerns regarding statistical power.

**Reply: We have revised the Methods and Results sections. The initial sample size calculation (n = 385) was based on prevalence estimates and assumptions consistent with probability sampling for national population. However, the present study was conducted using a convenience sampling approach, and recruitment was affected by cultural and social sensitivities related to biological sample collection among women. Although 149 individuals completed the questionnaire, only 133 participants met the eligibility criteria and provided fully matched urine samples suitable for analysis. As now clarified in the manuscript (Sample size calculation and Demographic Characteristics section), after applying eligibility criteria and confirming complete matching between biological samples and questionnaire data, the final analyzable cohort consisted of 133 participants.** **We acknowledge that the sample size achieved is smaller than the initially calculated target. This limitation has now been added as a future recommendation in the Conclusion sections. “**Future research incorporating larger and more geographically diverse populations is recommended to enhance statistical power and improve generalizability.”

5. Although a ROC curve is provided in the Supplementary Information (Figure S2), key diagnostic metrics remain insufficiently reported. The area under the curve, its confidence interval, and the criterion used to select the 7 ng/mL cutoff are not provided. In addition, Tables S5 and S6 show that nearly half of the 15 self-reported “smokers” are ex-smokers, several with undetectable cotinine levels, and at least one classified as passive exposure. This heterogeneity within the reference smoker group limits the validity and stability of the ROC analysis.

**Reply: Thanks a lot for this comment. We have clarified the ROC analysis and expanded the description of cutoff determination in the revised manuscript. Urinary cotinine (continuous variable) was evaluated against self-reported smoking status using ROC curve analysis. Sensitivity remained 100% across a wide range of thresholds (0.036–14 ng/mL), while specificity progressively increased with higher cutoff values, reaching 92% at 6.0 ng/mL, 95% at 6.8 ng/mL, and 97% at 14 ng/mL. Although 14 ng/mL provided the highest specificity while maintaining full sensitivity, thresholds above 6 ng/mL already demonstrated strong discriminatory performance. Therefore, a cutoff of 7 ng/mL was selected as a balanced and practical threshold for distinguishing smokers from nonsmokers in this population. The revised text now provides clearer justification for this selection.** **In the revised manuscript, we have now explicitly reported the area under the ROC curve (AUC) along with its 95% confidence interval to better demonstrate the overall diagnostic performance of urinary cotinine. The criterion used to select the 7 ng/mL cutoff has also been clarified. Specifically, the threshold was chosen based on maximizing discriminatory performance while maintaining 100% sensitivity and achieving high specificity (>90%), consistent with ROC-derived likelihood ratios and practical applicability for population-based classification. Regarding the heterogeneity within the self-reported smoker group, we acknowledge that some participants identified as smokers were ex-smokers or reported passive exposure, and a few had undetectable cotinine levels. To address this concern, we carefully re-examined the classification criteria and clarified in the manuscript that self-reported smoking status was used as the reference variable for ROC construction. We have also discussed this heterogeneity as a limitation, noting that inclusion of ex-smokers within the reference smoker group may affect ROC stability and slightly influence diagnostic estimates.**
6. The proposed 7 ng/mL cutoff does not adequately account for overlap between heavy passive exposure and light active smoking. Table 2, Table S2, and Figure 6 demonstrate substantial overlap between nonsmokers and passive smokers in a population with high household second-hand smoke exposure, increasing the risk of misclassification.

**Reply: The overlap between heavy passive exposure and light active smoking may occur when using urinary cotinine concentrations alone, particularly at lower cutoff thresholds. As shown in the ROC analysis, specificity increases with higher cutoff values, which helps reduce potential misclassification between these exposure categories. The selected 7 ng/mL cutoff was chosen to balance sensitivity and specificity while minimizing misclassification risk; however, we recognize that biochemical measures cannot always fully distinguish heavy second-hand exposure from light active smoking. This limitation has now been explicitly acknowledged in the revised manuscript, and we emphasize that cotinine levels should be interpreted alongside self-reported smoking behavior and exposure history for more accurate classification.**

7. Participants were recruited exclusively from hospital staff, patients, and university students. This convenience sampling frame does not reflect the general Saudi female population and introduces selection bias, limiting external validity. The manuscript does not acknowledge this limitation and incorrectly implies random sampling. This should be corrected and discussed.

**Reply: The Study Population and Setting section has been revised to clearly specify the recruitment locations and timeframe. Participants were recruited between May and December 2024 from King Saud Medical City (including patients attending the sample collection department), employees at King Khalid University Hospital, and students from various colleges of King Saud University (KSU). The inclusion criteria are now explicitly stated: Saudi females aged ≥18 years who agreed to participate, were able to complete the questionnaire, and provided a urine sample. These revisions improve clarity regarding the study setting, recruitment strategy, and eligibility criteria, thereby enhancing transparency and reproducibility.** **A limitation sentence was added to the conclusion** “A key limitation of this study was the difficulty in recruiting female participants for urine sample collection due to cultural and social sensitivities, which reduced the sample size and may limit generalizability.”

8. Several paragraphs in the Introduction provide extensive background on metabolomics and LC-MS/MS applications. While informative, some of this material does not directly advance the specific rationale of the present study. Streamlining this section to focus more clearly on the public health motivation and the application of LC-MS/MS for cotinine quantification would improve clarity.

**Reply: The introduction paragraph on LC-MS/MS application was revised to focus on focus the public health relevance of tobacco exposure assessment and the specific application of LC-MS/MS for urinary cotinine quantification.**

9. The study collected a rich set of demographic and behavioral variables. However, the statistical analysis is largely descriptive. Conducting multivariable analyses to assess independent predictors of urinary cotinine levels or smoking status would add depth to the results and enhance the overall robustness of the conclusions.

**Reply: We agree that multivariable analysis could provide additional insight into independent predictors of urinary cotinine levels or smoking status. However, the primary objective of this study was methodological namely, the validation of an LC-MS/MS method and the establishment of a population-specific cutoff value for urinary cotinine. Additionally, the relatively small sample size and the limited number of confirmed smokers reduced the statistical power required to perform reliable multivariable modeling without risking model overfitting and unstable estimates. Therefore, the analysis was intentionally focused on descriptive statistics and ROC-based diagnostic evaluation, which were aligned with the study objectives.**

10. The manuscript would benefit from clarification on whether the questionnaire was pilot-tested or validated prior to use. If these steps were undertaken, they should be reported. If not, acknowledging this as a limitation would improve transparency.

**Reply:** **The questionnaire was specifically developed to align with the objectives of the present study and was structured into nine clearly defined sections covering demographic characteristics, smoking status, smoking history, frequency and duration, quit attempts, second-hand smoke exposure, health impacts, and open-ended feedback. The questionnaire was reviewed internally by the research team to ensure clarity, relevance, and content appropriateness prior to implementation. However, a formal psychometric validation process was not conducted. We have also acknowledged this as a methodological limitation in the conclusion section.**

11. Urinary cotinine concentrations exhibit marked skewness and substantial divergence between means and medians (Table 2). Although group comparisons were performed using unpaired t-tests, variance tests indicate significant heteroscedasticity (Table 3). In this context, alternative approaches that do not assume equal variances (e.g., Welch’s t-test) or nonparametric or transformed analyses would provide more robust support for the reported group differences.

**Reply:** **Due to the study’s sample size and scope, we did not re-run alternative statistical analyses; however, the results remain descriptive and intended primarily to illustrate patterns in cotinine levels across groups. We have now acknowledged this limitation in the revised manuscript and recommend that future studies with larger cohorts consider Welch’s t-test, nonparametric tests, or data transformation to strengthen statistical robustness.**

12. The presence of extreme values among smokers and passive smokers suggests that some group differences may be influenced by a small number of high observations. Complementary median-based or nonparametric analyses would improve robustness.

**Reply:** **We have now acknowledged in the revised manuscript that extreme values and skewed distributions may affect mean-based comparisons, and we recommend that future studies with larger sample sizes incorporate median-based or nonparametric analyses to enhance statistical robustness.**

13. Because vaping is reported among participants, and nicotine delivery differs from combustible tobacco use, failure to account for vaping behavior may confound cotinine-based classification. This should be addressed analytically or acknowledged as a limitation.

**Reply:** **We acknowledge that vaping was reported among some participants and that nicotine delivery from electronic cigarettes may differ in pattern and intensity compared with combustible tobacco products. Because urinary cotinine reflects systemic nicotine exposure regardless of the source, it does not distinguish between combustible tobacco use and vaping. The primary aim of this study was to assess overall nicotine exposure using cotinine as a biomarker rather than to differentiate between nicotine delivery methods. However, we agree that the presence of vaping may introduce heterogeneity in exposure patterns and could potentially influence cotinine-based classification. This has now been explicitly acknowledged as a limitation in the revised manuscript. Future studies with larger cohorts should stratify analyses by product type (combustible vs. electronic) to better characterize exposure dynamics.**

14. Figure 4 is described as part of the LC-MS optimization. However, its contribution to the main quantitative and exposure analyses is limited. The authors may consider justifying its inclusion as a main figure or moving it to the Supplementary Information.

**Reply: Figure 4 was moved to supplementary.**

15. With only 15 self-reported smokers, the study has limited power to analyze smoking behaviors (Objective 4), and the ROC-based cutoff determination is statistically limited. This limitation should be explicitly acknowledged and conclusions tempered accordingly.

**Reply:** **We acknowledge that the relatively small number of self-reported smokers (n = 15) limits statistical power for detailed analysis of smoking behaviors (Objective 4) and may affect the stability and precision of the ROC-based cutoff determination. The primary aim of the study was methodological, focusing on biomarker validation and preliminary cutoff estimation within this specific population. We agree that the limited number of smokers constrains subgroup analyses and may influence the robustness of diagnostic performance estimates. This limitation has now been explicitly acknowledged in the revised manuscript, and the conclusions have been tempered to reflect that the proposed cutoff should be considered preliminary and requires confirmation in larger cohorts.**

**Minor comments**

Urinary cotinine concentrations are reported without correction for urine dilution (e.g., creatinine normalization). This should be justified or acknowledged as a limitation.

**Reply:** **We acknowledge that urinary cotinine concentrations were not corrected for urine dilution using creatinine normalization. This has now been explicitly stated in the manuscript as a limitation, and we note that future studies should consider creatinine-adjusted cotinine measurements to improve accuracy and comparability.**

The timing of urine collection relative to last nicotine exposure is not described and may influence cotinine levels. Therefore, clarification would improve interpretability.

**Reply:** **We agree that the timing of urine collection relative to last nicotine exposure may influence cotinine concentrations. Since the exact timing was not systematically recorded, this has now been clarified and acknowledged as a limitation in the revised manuscript.**

The criteria used to classify participants as “passive smokers” are not clearly defined and should be explicitly stated to improve reproducibility.

**Reply:** **The criteria used to classify participants as passive smokers have now been explicitly defined in the Methods section. Passive smokers were identified based on self-reported regular exposure to household or close-environment smokers despite not reporting active smoking.**
Inconsistencies between figure labels cited in the text (e.g., Figure 6E) and the corresponding figure captions should be corrected.

**Reply:** **All figure citations have been carefully reviewed and corrected to ensure consistency between in-text references and figure captions.**

Widespread language and clarity issues are present throughout the manuscript and, in several sections, hinder interpretation of methods and results. Substantive language editing is required (capitalization consistency, redundancy, punctuation and mixed US and UK English).

**Reply: The manuscript has undergone thorough language editing to improve clarity, reduce redundancy, standardize capitalization, ensure punctuation consistency, and harmonize English usage throughout the text.**

Reviewer #2: This manuscript reports a meaningful investigation of tobacco smoke exposure in Saudi women using urinary cotinine as a core biomarker. Overall, the study design is technically rigorous, the experimental procedures are well-controlled, and the presented data adequately support the primary conclusions. The statistical analyses have been implemented appropriately, including rigorous normality assessments, correct selection of parametric/non-parametric tests, and complete reporting of effect sizes and 95% confidence intervals, which meets PLOS ONE’s statistical rigor standards.

**Reply:** **We sincerely thank the reviewer for his thorough evaluation and constructive comments. We appreciate all the efforts made in reviewing our manuscript. We have carefully revised the manuscript to address all raised concerns.**

However, three critical revisions are required to achieve full compliance with the journal’s policies and enhance the scientific impact of the work, justifying the Major Revision decision:

Data Availability Compliance: The manuscript does not yet fully adhere to PLOS ONE’s Data Availability Policy. Raw data underlying urinary cotinine measurements, participant demographic data, and ROC analysis results for cutoff value determination have not been made publicly available. All individual data points supporting summary statistics must be deposited in a recognized public repository (e.g., Dryad, Figshare, Mendeley Data) with a persistent DOI. The Data Availability Statement in the revised manuscript must explicitly include the repository link, DOI, and any justified restrictions on data sharing (e.g., participant privacy protections for this human subjects study).

**Reply:** **We thank the reviewer for emphasizing adherence to PLOS ONE’s Data Availability Policy. The dataset contains sensitive human participant information, and although all direct identifiers have been removed, institutional ethical approval restricts unrestricted public deposition of the raw data. Therefore, the data cannot be made fully publicly available. However, de-identified data underlying the findings will be made available upon reasonable request to the corresponding author, subject to approval by the institutional ethics committee to ensure compliance with participant confidentiality protections. The revised manuscript now includes a clarified Data Availability Statement explaining these restrictions and the procedure for data access. The raw data was attached also for reviewing by the editor and reviewers.**

Ethical Consent Procedure Elaboration: The current ethical statement is insufficiently detailed for a human subjects study focused on a culturally specific population (Saudi women). Please supplement the Methods section with a comprehensive description of the informed consent process, including: (1) the mode of consent (written/verbal) and justification for the chosen mode (in line with local ethical guidelines); (2) how study objectives, risks, benefits, and data confidentiality protocols were communicated to participants (e.g., in Arabic, with translation verification); (3) provisions for vulnerable participants (if applicable); and (4) explicit confirmation that consent was obtained prior to any study procedures (sample collection and survey administration). Ensure the full IACUC/IRB approval number and the name of the reviewing ethical committee are clearly stated.

**Reply:** **In the revised manuscript, the Ethical Considerations section has been expanded to provide a comprehensive description of the consent process. The study was approved by the Institutional Review Board (IRB) of the College of Medicine, King Saud University (Reference No. E-24-8587). Electronic written informed consent was obtained from all participants prior to any study procedures, including completion of the questionnaire and urine sample collection. Participants were provided with a detailed explanation of the study objectives, procedures, potential risks, and anticipated benefits through an information sheet presented in Arabic to ensure full comprehension. Participation was entirely voluntary, and participants were informed of their right to withdraw at any time without consequences. Confidentiality measures have been clearly described, including the assignment of unique identification codes and the exclusion of personally identifiable information from the dataset. The study involved only Saudi females aged 18 years or older; therefore, no minors or legally vulnerable individuals were included.**

Update of Recent Literature: The introduction and discussion sections rely heavily on citations published before 2023, which limits the contextualization of this work within the latest advances in tobacco smoke exposure research. Please supplement the literature review with key publications from 2023–2026, focusing on: (1) contemporary trends in secondhand tobacco smoke exposure among women in the Middle East/North Africa region; (2) updated validation of urinary cotinine as a biomarker for tobacco smoke exposure in low-to-middle income countries; and (3) recent debates on cotinine cutoff values for distinguishing active vs. passive smoking in female populations. This update will strengthen the justification for the study’s research gap and highlight the novelty of your findings.

**Reply: The Introduction and Discussion have been updated to include recent literature (2023–2026)**
