# Supplementary material for: Biomarker-based assessment of tobacco smoke exposure in Saudi females via analysis of urinary cotinine level
Source: PLoS One. 2026 Aug 11;21(8):e0355166. doi: 10.1371/journal.pone.0355166 (PMC13460576; doi:10.1371/journal.pone.0355166)
Supplement: Supplementary file 3 [file pone.0355166.s004.docx]

**Author Reply List**

To

**Prof. Thiago P. Fernandes, PhD**

**Academic Editor**

**PLOS One**

First thanks for handling our manuscript and considering it for publication in PLOS ONE after careful revision. Kindly, find all our responses to the raised queries. The manuscript has been carefully revised.

 Review Comments to the Author

Reviewer #1: Most of the previous comments have been addressed in the revised manuscript. However, several issues remain only partially resolved and would benefit from further clarification or revision:

**Reply: Thanks a lot for the reviewer comments, we appreciate all the efforts made for the evaluation of our manuscript. The manuscript was revised and the raised points were addressed and we hope the updated version fulfils the reviewer’s requirements.**

Major point:

The authors indicate that alternative statistical analyses were not performed due to the study’s sample size and scope. However, the concern raised relates to the appropriateness of the statistical test used rather than the study scope. Because variance tests indicate significant heteroscedasticity (p < 0.0001), the assumptions of the standard unpaired t-test are violated. Under these conditions, a Welch’s t-test or a nonparametric test (e.g., Mann–Whitney) would be more appropriate. Simply acknowledging this as a limitation does not fully resolve the methodological concern, and small sample size does not justify the use of a statistical test whose assumptions are not satisfied. Because these statistical comparisons directly support the reported results, the choice of statistical test may influence the strength of the study’s conclusions. As such, the currently reported p-values may not be optimal.

**Reply:** **Thanks a lot for the reviewer’s comment,** **, the statistical analyses were revised to account for the significant heteroscedasticity observed in urinary cotinine concentrations. Brown–Forsythe and Bartlett’s tests confirmed unequal variances across smoking categories (p < 0.0001). Therefore, pairwise comparisons were re-evaluated using Welch’s corrected unpaired t-tests, which do not assume equal variances. Median and interquartile range (IQR) values were also added to better represent the skewed distribution and influence of extreme observations. The revised analyses supported the original interpretation while providing a more statistically robust assessment of group differences.**

Minor points:

1. Given the limited sample size and the convenience sampling design, the study appears exploratory in nature. The manuscript would benefit from clarifying whether it should be framed as a pilot or exploratory study and from tempering statements suggesting the establishment of a population-specific cutoff. The need for external validation in larger and more representative cohorts should be emphasized.

**Reply: The manuscript has been revised to clarify that the present work should be considered an exploratory/pilot biomarker study due to the modest sample size and convenience sampling design. Statements implying definitive establishment of a population-specific cutoff have been moderated throughout the manuscript. The proposed 7 ng/mL threshold is now described as a preliminary cutoff requiring validation in larger and more representative cohorts.**

2. The ROC analysis should report the Area Under the Curve (AUC) with its 95% confidence interval, as these are standard measures of diagnostic performance.
Example:

• Area under the ROC curve (AUC) = 0.94

• 95% CI = 0.88–0.99

• p < 0.001

**Reply: The ROC analysis section has been revised to include the Area Under the Curve (AUC), corresponding 95% confidence interval, and p-value as standard indicators of diagnostic performance. These values are now explicitly reported in both the Results section and the figure legend for improved transparency and interpretability.**

3. Given the pronounced skewness and the presence of extreme values in urinary cotinine concentrations (Table 2 and Figure 5), mean-based comparisons may not fully represent the underlying distribution. Reporting median and interquartile range (IQR) values and/or performing nonparametric statistical analyses (e.g., Mann–Whitney test) would provide a more robust comparison between groups. Acknowledging this only as a future direction does not fully address this.
**Reply: We appreciate the reviewer’s suggestion, median and interquartile range (IQR) values for urinary cotinine concentrations have now been added to the Results section and relevant tables to better represent the skewed distribution of the data.**

4. Because the ROC analysis is based on a relatively small number of smokers, the interpretation of diagnostic performance should be moderated. Describing the results as demonstrating “excellent diagnostic performance” may be overly strong. The proposed cutoff may be better described as preliminary and requiring validation in larger cohorts.

**Reply: The wording describing the ROC performance has been revised and moderated throughout the manuscript. Rather than describing the diagnostic performance as “excellent,” the manuscript now states that the ROC analysis demonstrated “good preliminary discriminatory performance.” We also emphasize that the proposed cutoff value should be considered preliminary and requires external validation in larger and more representative populations.**

Reviewer #3: Thank you for the opportunity to review the manuscript. I consider the topic relevant and recognize the research's potential to contribute to the scientific development of the field. However, some methodological issues deserve attention, as highlighted below:

**Reply: Thanks a lot for the reviewer comments, we appreciate all the efforts made for the evaluation of our manuscript. The manuscript was revised and the raised points were addressed, and we hope the updated version fulfils the reviewer’s requirements.**

At certain points, the text becomes verbose and confusing. For example, at the end of the introduction, the authors justify the research and present the objective repetitively, reiterating this information more than once. It is suggested that the introduction be reorganized in a funnel format, starting with more general aspects of the topic, moving on to more specific elements, and ending with a clear presentation of the study's objective and hypotheses. Therefore, a structural review of the text is recommended to make it more concise, fluid, and objective.

**Reply:** **The Introduction has been substantially reorganized following a funnel structure, beginning with the broader public health significance of tobacco exposure, followed by the role of urinary cotinine and LC-MS/MS analysis, and concluding with the specific rationale and objectives of the present study.**

In the methods section, it is suggested that the authors provide more detailed information about the procedures adopted in the study. It is recommended to specify the location where the research was conducted, the laboratory or institution responsible for analyzing the samples, as well as the professionals involved in collecting and evaluating these samples. Furthermore, it would be important to clarify how the recruitment sites were defined, indicating whether the selection occurred by convenience or through other methodological criteria. A clearer description of the study population is also suggested, including its main characteristics and eligibility criteria, in order to facilitate understanding of the methodological design and the reproducibility of the research.

**Reply:** **We appreciate the reviewer’s recommendation. The Methods section has been revised to provide more detailed information regarding the study setting, recruitment process, eligibility criteria, and sample collection procedures. The manuscript now clearly states that participants were recruited by convenience sampling from King Saud Medical City, King Khalid University Hospital employees, and students from King Saud University between May and December 2024. Additional details regarding the laboratory analysis setting and urine sample processing procedures were also added to improve reproducibility and methodological transparency.**

In the section regarding sample size calculation, the authors report that it was not possible to reach the estimated sample size. However, in a previous section, the statement remains that the data collection was conducted until the N suggested by the sample size calculation was reached, which generates inconsistency in the methodological description. It is suggested that this information be reviewed and harmonized to ensure greater clarity and coherence in the text.

**Reply:** **We thank the reviewer for identifying this inconsistency. The sample size calculation section has been revised and harmonized throughout the manuscript. The revised text now clearly explains that although the calculated target sample size was 385 participants, recruitment challenges and eligibility criteria resulted in a final analyzable sample of 133 participants. Statements implying that the target sample size was fully achieved were removed for consistency and clarity.**

It is also suggested that the authors describe the sample recruitment process in more detail, clarifying, for example, whether the selection occurred by convenience and how the participants had access to information about the research. It is important to specify how these women were recruited, what dissemination strategies were used, and in what contexts the participants were recruited. Furthermore, some of the highlighted information is scattered throughout the text, in sections that do not adequately correspond to the content presented. It is recommended to reorganize this information into a single appropriate topic or subsection, in order to promote clarity, methodological organization, and reader comprehension.

**Reply:** **The Sampling Technique subsection has been revised to clarify that convenience sampling was employed. Additional details describing recruitment procedures and dissemination strategies have also been added. Eligible participants from hospitals and universities in Riyadh were approached directly and invited to participate voluntarily after receiving information regarding the study objectives and procedures.**

The last sentence of the section regarding the sample size calculation was unclear and, as written, compromises the understanding of the passage. It is suggested that its wording be revised or relocated to another section of the manuscript where the information can be better contextualized.

**Reply:** **The sample size section has been revised and simplified to improve clarity and contextual coherence. The sentence referring to the Saudi female population size was rewritten and integrated appropriately within the sample size rationale.**

It is also recommended that the authors provide more detailed information about the questionnaire used in the study, specifying whether the instrument underwent a validation or adaptation process or whether it was developed based on previously existing scientific literature. This information is important to strengthen the methodological quality and reliability of the data obtained.

**Reply:** **Additional information regarding questionnaire development has been incorporated into the revised manuscript. The questionnaire was specifically designed to address the objectives of the study based on relevant literature and smoking exposure variables. The manuscript now also clarifies that, although the questionnaire was reviewed for clarity and relevance, it did not undergo formal psychometric validation, which has been acknowledged as a study limitation.**

Furthermore, the methodological description implied that the Informed Consent Form was included as part of the questionnaire. If this interpretation is correct, clarification is necessary, since, ethically, participants must have access to the ICF before the start of the research and prior to submitting to any data collection instrument. It is suggested that the authors review the description of this procedure to avoid ambiguities and ensure greater clarity regarding the ethical aspects of the study.

**Reply:** **The Ethical Considerations section has been revised to clarify that participants were first provided with a detailed participant information sheet explaining the study objectives, procedures, confidentiality measures, risks, and benefits before participation. Electronic written informed consent was obtained prior to questionnaire completion and urine sample collection. The revised wording eliminates ambiguity regarding the consent process.**

In my perception, the description of the sampling technique should be included in the section referring to the sample, since it is information directly related to the composition and selection of the study participants. As it is currently presented, after the description of the instruments, the information becomes decontextualized and loses its methodological function. This aspect reinforces the perception of structural disorganization in the text, highlighting the need for revision and reorganization of the methodological sections, in order to promote greater clarity, coherence, and fluency in reading the manuscript. Throughout the reading of the manuscript, after the description of the instruments used, I felt the lack of a more detailed explanation about the methods employed for the evaluation of urine samples. Later, I realized that this information is described in another section of the text and in an appropriate way. However, it is suggested that this information be reorganized, positioning it immediately after the instruments section, since it refers directly to the techniques and procedures for data collection.

**Reply: The Methods section has been reorganized to improve logical flow. The urine sample preparation and LC-MS/MS analytical procedures are now positioned directly after the questionnaire and sampling descriptions, facilitating better methodological continuity.**

At times, the writing of the manuscript presents characteristics closer to a research project than to a scientific article proper. As an example, the inclusion of extensive information on the risks and benefits of the research stands out, an aspect that, depending on the journal's guidelines, may require a more objective and synthetic approach. In addition, if the authors choose to maintain the description of the risks involved, greater methodological detail is suggested. The text states that the research offered minimal risks to the participants, but does not specify what these risks would be, nor does it describe the strategies adopted to minimize them. It is recommended to clarify these aspects in order to give greater ethical and methodological consistency to the manuscript.

**Reply:** **The manuscript has undergone substantial language and structural editing to improve conciseness and align the presentation with standard scientific article formatting. Excessively detailed descriptions regarding risks and benefits were shortened and streamlined.** **The Ethical Considerations section has been revised to clarify that the study involved minimal risk due to non-invasive urine sample collection and completion of a self-administered questionnaire. Confidentiality protections, anonymization procedures, and voluntary participation rights are now described more clearly to demonstrate the measures adopted to minimize potential risks.**

On line 267, the authors reiterate the study's objective, information that had already been clearly presented at the end of the introduction. This repetition can also be observed at other points in the manuscript, contributing to redundant and less fluid writing. Therefore, a general review of the writing is suggested, in order to make the text more objective, cohesive, and concise, avoiding unnecessary repetitions throughout the sections.

**Reply:** **Repeated statements of the study objectives throughout the manuscript have been removed or condensed. The manuscript was carefully revised to improve cohesion, reduce redundancy, and enhance readability.**

It is suggested that the classification of smoking status be presented before the topic concerning data integration. This is because the data integration section establishes a more direct connection with the data analysis stage and should therefore be positioned in logical sequence to the analyses performed. The current organization compromises methodological fluidity and makes it difficult to understand the structure of the study design. It is recommended to reorganize these subsections in order to favor greater coherence and chaining of the information presented.

**Reply:** **The subsections within the methodological workflow have been reorganized.**

It was not clear in the manuscript whether the analysis of the data distribution was performed prior to the application of statistical tests. Considering that parametric tests, such as ANOVA and t-tests, were used, it is suggested that the authors report whether the assumptions necessary for these analyses were verified, especially regarding the normality of the data distribution and the homogeneity of variances. If these analyses were conducted, it is recommended to describe the procedures adopted and the tests used for this verification.

**Reply:** **Additional details were added to the Statistical Analysis subsection. The revised manuscript now states that data distribution and homogeneity of variances were assessed prior to inferential analyses. Because urinary cotinine concentrations demonstrated skewed distributions and significant heteroscedasticity, Welch’s corrected unpaired t-test was applied for pairwise comparisons. Median and interquartile range (IQR) values were additionally reported to better characterize non-normal distributions and reduce the influence of extreme values.**

In the discussion section, I felt the lack of a more in-depth articulation with the literature in the area, especially in the sense of comparing the study's findings with previously published evidence, corroborating or contradicting them when pertinent. The inclusion of this discussion is important to strengthen the interpretation of the results and situate the study's contribution in the scientific field.

**Reply:** **The Discussion section has been revised to strengthen comparison with previously published studies. Additional recent literature related to urinary cotinine biomarkers, passive smoking exposure, and cutoff determination has been incorporated to contextualize our findings and highlight similarities and differences with prior reports.**
In addition, it is considered that the limitations section would be more appropriately positioned at the end of the discussion, and not in the conclusion. The conclusion should prioritize the synthesis of the main findings and implications of the study, avoiding the insertion of new analytical elements.

**Reply:** **The limitations discussion has now been moved from the Conclusion section to the end of the Discussion section to improve manuscript structure and align with standard scientific writing convention.**

Finally, it is suggested that, in addition to describing the limitations of the research, the authors also clarify which methodological strategies were adopted to minimize its potential impacts. This discussion can strengthen the robustness of the study and demonstrate greater scientific rigor in conducting the research.

**Reply:** **Additional statements were included in the Discussion section to clarify measures undertaken to strengthen study rigor despite the acknowledged limitations. These include the use of validated LC-MS/MS methodology, biochemical verification alongside self-reported data, standardized sample processing, and application of variance-robust statistical analyses.**
