# Supplementary material for: Biomarker-based assessment of tobacco smoke exposure in Saudi females via analysis of urinary cotinine level
Source: PLoS One. 2026 Aug 11;21(8):e0355166. doi: 10.1371/journal.pone.0355166 (PMC13460576; doi:10.1371/journal.pone.0355166)
Supplement: Supplementary file 4 [file pone.0355166.s005.docx]

**Author Reply List**

To

**Prof. Thiago P. Fernandes, PhD**

**Academic Editor**

**PLOS One**

First thanks for handling our manuscript and considering it for publication in PLOS ONE after careful revision. Kindly, find all our responses to the raised queries. The manuscript has been carefully revised.

 Review Comments to the Author

Reviewer #1: The authors have addressed several of the previous comments. However, the main statistical concern remains still only partially addressed.

**Reply: Thanks a lot for the reviewers’ comments, we appreciate all the efforts made to the evaluation of our manuscript. We appreciate the efforts of the reviewer in the improvement of our manuscript. The manuscript was revised and the comments raised were addressed, and we hope the updated versions meet the reviewer requirements.**

Although the manuscript now states that Welch’s corrected unpaired t-tests were used, the values reported in Table 3 appear inconsistent with Welch’s test. The reported degrees of freedom, such as t(70), t(74), and t(116), correspond exactly to the standard equal-variance unpaired t-test calculation, n1 + n2 - 2. Since the variance tests showed significant heteroscedasticity, the authors should report the actual Welch-adjusted degrees of freedom, confidence intervals, and p-values.

**Reply:** **Thanks a lot for the reviewer’s comment, Table 3 was corrected to report the appropriate Welch-adjusted degrees of freedom, 95% confidence intervals, t statistics, and corresponding p-values generated by GraphPad Prism. These revisions do not alter the interpretation of the results, and the significant differences between smokers and the other study groups remain unchanged.**

The authors should also clarify the inconsistency in group sizes. The manuscript states that the refined classification included 11 smokers, 62 passive smokers, and 60 nonsmokers, whereas Tables 2 and 3 report 15 smokers, 61 passive smokers, and 57 nonsmokers. The classification used for each analysis should be clearly stated and applied consistently across the Results, tables, figures, and ROC analysis.

**Reply:** **The discrepancy resulted from presenting two different participant classifications. Specifically, the ROC analysis used the self-reported smoking status (15 self-reported smokers) as the reference classification, whereas the biomarker-based classification applied the urinary cotinine cutoff to categorize participants according to measured cotinine concentrations. The final statistical analyses were consistently performed using the complete study dataset comprising 15 smokers, 61 passive smokers, and 57 nonsmokers (n = 133). The manuscript has been carefully revised to ensure that these group sizes are reported consistently throughout the Results, Tables, Figures, and ROC analysis.**

Finally, the authors should briefly explain how zero or non-detected cotinine values were handled in the logarithmic-scale plots.

**Reply: A brief explanation has now been added to the Statistical Analysis section and to the figure 5 legend. Samples with non-detectable urinary cotinine concentrations were assigned a small positive value corresponding to the lower detection limit solely for visualization on logarithmic-scale graphs. This adjustment was applied only for graphical presentation and was not used in any statistical analyses, which were performed using the original measured values.**
