# Supplementary material for: Biomarker-based assessment of tobacco smoke exposure in Saudi females via analysis of urinary cotinine level
Source: PLoS One. 2026 Aug 11;21(8):e0355166. doi: 10.1371/journal.pone.0355166 (PMC13460576; doi:10.1371/journal.pone.0355166)
Supplement: Peer review history

# Peer review: Decision Letter 0

**DOI:** 10.1371/journal.pone.0355166.r001

**Authors:** Thiago Fernandes

24 Feb 2026

PONE-D-25-62265

Biomarker-based Assessment of Tobacco Smoke Exposure in Saudi Females via analysis of Urinary Cotinine level

PLOS One

Dear Dr. Alanazi,

Thank you for submitting your manuscript to PLOS ONE. After careful consideration, we feel that it has merit but does not fully meet PLOS ONE’s publication criteria as it currently stands. Therefore, we invite you to submit a revised version of the manuscript that addresses the points raised during the review process.

==============================

Please respond to all comments and highlight the changes in the revised manuscript.

==============================

Please submit your revised manuscript by Apr 10 2026 11:59PM. If you will need more time than this to complete your revisions, please reply to this message or contact the journal office at plosone@plos.org. When you're ready to submit your revision, log on to https://www.editorialmanager.com/pone/ and select the 'Submissions Needing Revision' folder to locate your manuscript file.

Please include the following items when submitting your revised manuscript:

- A letter that responds to each point raised by the academic editor and reviewer(s). You should upload this letter as a separate file labeled 'Response to Reviewers'.
- A marked-up copy of your manuscript that highlights changes made to the original version. You should upload this as a separate file labeled 'Revised Manuscript with Track Changes'.
- An unmarked version of your revised paper without tracked changes. You should upload this as a separate file labeled 'Manuscript'.

If you would like to make changes to your financial disclosure, please include your updated statement in your cover letter. Guidelines for resubmitting your figure files are available below the reviewer comments at the end of this letter.

If applicable, we recommend that you deposit your laboratory protocols in protocols.io to enhance the reproducibility of your results. Protocols.io assigns your protocol its own identifier (DOI) so that it can be cited independently in the future. For instructions see: https://journals.plos.org/plosone/s/submission-guidelines#loc-laboratory-protocols. Additionally, PLOS ONE offers an option for publishing peer-reviewed Lab Protocol articles, which describe protocols hosted on protocols.io. Read more information on sharing protocols at https://plos.org/protocols?utm_medium=editorial-email&utm_source=authorletters&utm_campaign=protocols.

We look forward to receiving your revised manuscript.

Kind regards,

Thiago P. Fernandes, PhD

Academic Editor

PLOS One

Journal Requirements:

When submitting your revision, we need you to address these additional requirements.

1. Please ensure that your manuscript meets PLOS ONE's style requirements, including those for file naming. The PLOS ONE style templates can be found at

https://journals.plos.org/plosone/s/file?id=wjVg/PLOSOne_formatting_sample_main_body.pdf and https://journals.plos.org/plosone/s/file?id=ba62/PLOSOne_formatting_sample_title_authors_affiliations.pdf

2. Your ethics statement should only appear in the Methods section of your manuscript. If your ethics statement is written in any section besides the Methods, please delete it from any other section.

3. Please amend the manuscript submission data (via Edit Submission) to include author Alaa Abdulelah Alansari, Haya I. Aljohar, Ghadeer Alotaibi, Nouf AL-Tuwayr, Muneerah Essa Alsuwaidan, Khetam Mosfer Alotaibi, Hayat Shaibah, Sultan M. Alghadeer, Hala Aldahshan , Afnan Bakhsh, Bandar Alghanem, Sana Saeed Alqarni

4. Your abstract cannot contain citations. Please only include citations in the body text of the manuscript, and ensure that they remain in ascending numerical order on first mention.

5. Please include a separate caption for each figure in your manuscript.

6. We note that there is identifying data in the Supporting Information file <E-24-8587_Ms. Alaa Abdulelah Alansari _Approval Letter.pdf>. Due to the inclusion of these potentially identifying data, we have removed this file from your file inventory. Prior to sharing human research participant data, authors should consult with an ethics committee to ensure data are shared in accordance with participant consent and all applicable local laws.

Data sharing should never compromise participant privacy. It is therefore not appropriate to publicly share personally identifiable data on human research participants. The following are examples of data that should not be shared:

-Name, initials, physical address

-Ages more specific than whole numbers

-Internet protocol (IP) address

-Specific dates (birth dates, death dates, examination dates, etc.)

-Contact information such as phone number or email address

-Location data

-ID numbers that seem specific (long numbers, include initials, titled “Hospital ID”) rather than random (small numbers in numerical order)

Data that are not directly identifying may also be inappropriate to share, as in combination they can become identifying. For example, data collected from a small group of participants, vulnerable populations, or private groups should not be shared if they involve indirect identifiers (such as sex, ethnicity, location, etc.) that may risk the identification of study participants.

Additional guidance on preparing raw data for publication can be found in our Data Policy (https://journals.plos.org/plosone/s/data-availability#loc-human-research-participant-data-and-other-sensitive-data) and in the following article: http://www.bmj.com/content/340/bmj.c181.long.

Please remove or anonymize all personal information (<specific identifying information in file to be removed>), ensure that the data shared are in accordance with participant consent, and re-upload a fully anonymized data set. Please note that spreadsheet columns with personal information must be removed and not hidden as all hidden columns will appear in the published file.

7. Please include captions for your Supporting Information files at the end of your manuscript, and update any in-text citations to match accordingly. Please see our Supporting Information guidelines for more information: http://journals.plos.org/plosone/s/supporting-information.

8. If the reviewer comments include a recommendation to cite specific previously published works, please review and evaluate these publications to determine whether they are relevant and should be cited. There is no requirement to cite these works unless the editor has indicated otherwise.

[Note: HTML markup is below. Please do not edit.]

Reviewers' comments:

Reviewer's Responses to Questions

**Comments to the Author**

1. Is the manuscript technically sound, and do the data support the conclusions?

The manuscript must describe a technically sound piece of scientific research with data that supports the conclusions. Experiments must have been conducted rigorously, with appropriate controls, replication, and sample sizes. The conclusions must be drawn appropriately based on the data presented.

Reviewer #1: Partly

Reviewer #2: Yes

**********

2. Has the statistical analysis been performed appropriately and rigorously?

Reviewer #1: No

Reviewer #2: Yes

**********

3. Have the authors made all data underlying the findings in their manuscript fully available?

The PLOS Data policy requires authors to make all data underlying the findings described in their manuscript fully available without restriction, with rare exception (please refer to the Data Availability Statement in the manuscript PDF file). The data should be provided as part of the manuscript or its supporting information, or deposited to a public repository. For example, in addition to summary statistics, the data points behind means, medians and variance measures should be available. If there are restrictions on publicly sharing data—e.g. participant privacy or use of data from a third party—those must be specified.

Reviewer #1: Yes

Reviewer #2: No

**********

4. Is the manuscript presented in an intelligible fashion and written in standard English?

PLOS ONE does not copyedit accepted manuscripts, so the language in submitted articles must be clear, correct, and unambiguous. Any typographical or grammatical errors should be corrected at revision, so please note any specific errors here.

Reviewer #1: No

Reviewer #2: Yes

**********

5. Review Comments to the Author

Please use the space provided to explain your answers to the questions above. You may also include additional comments for the author, including concerns about dual publication, research ethics, or publication ethics. (Please upload your review as an attachment if it exceeds 20,000 characters)

Reviewer #1: This manuscript addresses an important public health issue by combining biomarker-based assessment with self-reported data to evaluate tobacco exposure among Saudi females. While the study has merit, several methodological, statistical, and reporting issues need to be addressed before the conclusions can be adequately supported.

1. The manuscript contains multiple citation-formatting errors, including inconsistent use of superscripts (e.g., Lines 61, 62, 131, 133, 191, 215, and 220). Several in-text citations do not correspond to the reference list. Reference formatting should be corrected throughout to comply with journal guidelines.

2. The statement regarding underreporting of smoking among Saudi females due to cultural and social stigma (Lines 69-72) is not adequately supported by the cited references. Reference 5 reports prevalence among medical students but does not address stigma or underreporting, while Reference 6 discusses self-report bias in general populations without Saudi- or female-specific evidence. Consider citing https://smj.org.sa/content/40/9/867

3. References 8 and 9 appear to cover overlapping content and should be reviewed for redundancy.

4. The Methods section states that a minimum sample size of 385 participants was calculated and that recruitment continued until this target was reached. However, the final analyzed sample comprises only 133 participants. This discrepancy is not explained and raises concerns regarding statistical power.

5. Although a ROC curve is provided in the Supplementary Information (Figure S2), key diagnostic metrics remain insufficiently reported. The area under the curve, its confidence interval, and the criterion used to select the 7 ng/mL cutoff are not provided. In addition, Tables S5 and S6 show that nearly half of the 15 self-reported “smokers” are ex-smokers, several with undetectable cotinine levels, and at least one classified as passive exposure. This heterogeneity within the reference smoker group limits the validity and stability of the ROC analysis.

6. The proposed 7 ng/mL cutoff does not adequately account for overlap between heavy passive exposure and light active smoking. Table 2, Table S2, and Figure 6 demonstrate substantial overlap between nonsmokers and passive smokers in a population with high household second-hand smoke exposure, increasing the risk of misclassification.

7. Participants were recruited exclusively from hospital staff, patients, and university students. This convenience sampling frame does not reflect the general Saudi female population and introduces selection bias, limiting external validity. The manuscript does not acknowledge this limitation and incorrectly implies random sampling. This should be corrected and discussed.

8. Several paragraphs in the Introduction provide extensive background on metabolomics and LC-MS/MS applications. While informative, some of this material does not directly advance the specific rationale of the present study. Streamlining this section to focus more clearly on the public health motivation and the application of LC-MS/MS for cotinine quantification would improve clarity.

9. The study collected a rich set of demographic and behavioral variables. However, the statistical analysis is largely descriptive. Conducting multivariable analyses to assess independent predictors of urinary cotinine levels or smoking status would add depth to the results and enhance the overall robustness of the conclusions.

10. The manuscript would benefit from clarification on whether the questionnaire was pilot-tested or validated prior to use. If these steps were undertaken, they should be reported. If not, acknowledging this as a limitation would improve transparency.

11. Urinary cotinine concentrations exhibit marked skewness and substantial divergence between means and medians (Table 2). Although group comparisons were performed using unpaired t-tests, variance tests indicate significant heteroscedasticity (Table 3). In this context, alternative approaches that do not assume equal variances (e.g., Welch’s t-test) or nonparametric or transformed analyses would provide more robust support for the reported group differences.

12. The presence of extreme values among smokers and passive smokers suggests that some group differences may be influenced by a small number of high observations. Complementary median-based or nonparametric analyses would improve robustness.

13. Because vaping is reported among participants, and nicotine delivery differs from combustible tobacco use, failure to account for vaping behavior may confound cotinine-based classification. This should be addressed analytically or acknowledged as a limitation.

14. Figure 4 is described as part of the LC-MS optimization. However, its contribution to the main quantitative and exposure analyses is limited. The authors may consider justifying its inclusion as a main figure or moving it to the Supplementary Information.

15. With only 15 self-reported smokers, the study has limited power to analyze smoking behaviors (Objective 4), and the ROC-based cutoff determination is statistically limited. This limitation should be explicitly acknowledged and conclusions tempered accordingly.

Minor comments

Urinary cotinine concentrations are reported without correction for urine dilution (e.g., creatinine normalization). This should be justified or acknowledged as a limitation.

The timing of urine collection relative to last nicotine exposure is not described and may influence cotinine levels. Therefore, clarification would improve interpretability.

The criteria used to classify participants as “passive smokers” are not clearly defined and should be explicitly stated to improve reproducibility.

Inconsistencies between figure labels cited in the text (e.g., Figure 6E) and the corresponding figure captions should be corrected.

Widespread language and clarity issues are present throughout the manuscript and, in several sections, hinder interpretation of methods and results. Substantive language editing is required (capitalization consistency, redundancy, punctuation and mixed US and UK English).

Reviewer #2: This manuscript reports a meaningful investigation of tobacco smoke exposure in Saudi women using urinary cotinine as a core biomarker. Overall, the study design is technically rigorous, the experimental procedures are well-controlled, and the presented data adequately support the primary conclusions. The statistical analyses have been implemented appropriately, including rigorous normality assessments, correct selection of parametric/non-parametric tests, and complete reporting of effect sizes and 95% confidence intervals, which meets PLOS ONE’s statistical rigor standards.

However, three critical revisions are required to achieve full compliance with the journal’s policies and enhance the scientific impact of the work, justifying the Major Revision decision:

Data Availability Compliance: The manuscript does not yet fully adhere to PLOS ONE’s Data Availability Policy. Raw data underlying urinary cotinine measurements, participant demographic data, and ROC analysis results for cutoff value determination have not been made publicly available. All individual data points supporting summary statistics must be deposited in a recognized public repository (e.g., Dryad, Figshare, Mendeley Data) with a persistent DOI. The Data Availability Statement in the revised manuscript must explicitly include the repository link, DOI, and any justified restrictions on data sharing (e.g., participant privacy protections for this human subjects study).

Ethical Consent Procedure Elaboration: The current ethical statement is insufficiently detailed for a human subjects study focused on a culturally specific population (Saudi women). Please supplement the Methods section with a comprehensive description of the informed consent process, including: (1) the mode of consent (written/verbal) and justification for the chosen mode (in line with local ethical guidelines); (2) how study objectives, risks, benefits, and data confidentiality protocols were communicated to participants (e.g., in Arabic, with translation verification); (3) provisions for vulnerable participants (if applicable); and (4) explicit confirmation that consent was obtained prior to any study procedures (sample collection and survey administration). Ensure the full IACUC/IRB approval number and the name of the reviewing ethical committee are clearly stated.

Update of Recent Literature: The introduction and discussion sections rely heavily on citations published before 2023, which limits the contextualization of this work within the latest advances in tobacco smoke exposure research. Please supplement the literature review with key publications from 2023–2026, focusing on: (1) contemporary trends in secondhand tobacco smoke exposure among women in the Middle East/North Africa region; (2) updated validation of urinary cotinine as a biomarker for tobacco smoke exposure in low-to-middle income countries; and (3) recent debates on cotinine cutoff values for distinguishing active vs. passive smoking in female populations. This update will strengthen the justification for the study’s research gap and highlight the novelty of your findings.

**********

6. PLOS authors have the option to publish the peer review history of their article (what does this mean?). If published, this will include your full peer review and any attached files.

If you choose “no”, your identity will remain anonymous but your review may still be made public.

**Do you want your identity to be public for this peer review?**  For information about this choice, including consent withdrawal, please see our Privacy Policy.

Reviewer #1: No

Reviewer #2: No

**********

[NOTE: If reviewer comments were submitted as an attachment file, they will be attached to this email and accessible via the submission site. Please log into your account, locate the manuscript record, and check for the action link "View Attachments". If this link does not appear, there are no attachment files.]

To ensure your figures meet our technical requirements, please review our figure guidelines: https://journals.plos.org/plosone/s/figures

You may also use PLOS’s free figure tool, NAAS, to help you prepare publication quality figures: https://journals.plos.org/plosone/s/figures#loc-tools-for-figure-preparation.

NAAS will assess whether your figures meet our technical requirements by comparing each figure against our figure specifications.

# Author comment: Author response to Decision Letter 1

**DOI:** 10.1371/journal.pone.0355166.r002

3 Mar 2026

Author Reply List

To

Prof. Thiago P. Fernandes, PhD

Academic Editor

PLOS One

First thanks for handling our manuscript and considering it for publication in PLOS ONE after careful revision. Kindly, find all our responses to the raised queries. The manuscript has been carefully revised.

Review Comments to the Author

Reviewer #1: This manuscript addresses an important public health issue by combining biomarker-based assessment with self-reported data to evaluate tobacco exposure among Saudi females. While the study has merit, several methodological, statistical, and reporting issues need to be addressed before the conclusions can be adequately supported.

Reply: We sincerely thank the reviewer for his thorough evaluation and constructive comments. We appreciate all the efforts made in reviewing our manuscript. We have carefully revised the manuscript to address all raised concerns.

1. The manuscript contains multiple citation-formatting errors, including inconsistent use of superscripts (e.g., Lines 61, 62, 131, 133, 191, 215, and 220). Several in-text citations do not correspond to the reference list. Reference formatting should be corrected throughout to comply with journal guidelines.

Reply: All in-text citations have been carefully revised to comply with journal formatting guidelines.

2. The statement regarding underreporting of smoking among Saudi females due to cultural and social stigma (Lines 69-72) is not adequately supported by the cited references. Reference 5 reports prevalence among medical students but does not address stigma or underreporting, while Reference 6 discusses self-report bias in general populations without Saudi- or female-specific evidence. Consider citing https://smj.org.sa/content/40/9/867

Reply: We have revised the relevant paragraph and appropriate references were added, including the suggested article from the Saudi Medical Journal.

3. References 8 and 9 appear to cover overlapping content and should be reviewed for redundancy.

Reply: We reviewed both references and removed redundancy. One citation was deleted.

4. The Methods section states that a minimum sample size of 385 participants was calculated and that recruitment continued until this target was reached. However, the final analyzed sample comprises only 133 participants. This discrepancy is not explained and raises concerns regarding statistical power.

Reply: We have revised the Methods and Results sections. The initial sample size calculation (n = 385) was based on prevalence estimates and assumptions consistent with probability sampling for national population. However, the present study was conducted using a convenience sampling approach, and recruitment was affected by cultural and social sensitivities related to biological sample collection among women. Although 149 individuals completed the questionnaire, only 133 participants met the eligibility criteria and provided fully matched urine samples suitable for analysis. As now clarified in the manuscript (Sample size calculation and Demographic Characteristics section), after applying eligibility criteria and confirming complete matching between biological samples and questionnaire data, the final analyzable cohort consisted of 133 participants. We acknowledge that the sample size achieved is smaller than the initially calculated target. This limitation has now been added as a future recommendation in the Conclusion sections. “Future research incorporating larger and more geographically diverse populations is recommended to enhance statistical power and improve generalizability.”

5. Although a ROC curve is provided in the Supplementary Information (Figure S2), key diagnostic metrics remain insufficiently reported. The area under the curve, its confidence interval, and the criterion used to select the 7 ng/mL cutoff are not provided. In addition, Tables S5 and S6 show that nearly half of the 15 self-reported “smokers” are ex-smokers, several with undetectable cotinine levels, and at least one classified as passive exposure. This heterogeneity within the reference smoker group limits the validity and stability of the ROC analysis.

Reply: Thanks a lot for this comment. We have clarified the ROC analysis and expanded the description of cutoff determination in the revised manuscript. Urinary cotinine (continuous variable) was evaluated against self-reported smoking status using ROC curve analysis. Sensitivity remained 100% across a wide range of thresholds (0.036–14 ng/mL), while specificity progressively increased with higher cutoff values, reaching 92% at 6.0 ng/mL, 95% at 6.8 ng/mL, and 97% at 14 ng/mL. Although 14 ng/mL provided the highest specificity while maintaining full sensitivity, thresholds above 6 ng/mL already demonstrated strong discriminatory performance. Therefore, a cutoff of 7 ng/mL was selected as a balanced and practical threshold for distinguishing smokers from nonsmokers in this population. The revised text now provides clearer justification for this selection. In the revised manuscript, we have now explicitly reported the area under the ROC curve (AUC) along with its 95% confidence interval to better demonstrate the overall diagnostic performance of urinary cotinine. The criterion used to select the 7 ng/mL cutoff has also been clarified. Specifically, the threshold was chosen based on maximizing discriminatory performance while maintaining 100% sensitivity and achieving high specificity (>90%), consistent with ROC-derived likelihood ratios and practical applicability for population-based classification. Regarding the heterogeneity within the self-reported smoker group, we acknowledge that some participants identified as smokers were ex-smokers or reported passive exposure, and a few had undetectable cotinine levels. To address this concern, we carefully re-examined the classification criteria and clarified in the manuscript that self-reported smoking status was used as the reference variable for ROC construction. We have also discussed this heterogeneity as a limitation, noting that inclusion of ex-smokers within the reference smoker group may affect ROC stability and slightly influence diagnostic estimates.

6. The proposed 7 ng/mL cutoff does not adequately account for overlap between heavy passive exposure and light active smoking. Table 2, Table S2, and Figure 6 demonstrate substantial overlap between nonsmokers and passive smokers in a population with high household second-hand smoke exposure, increasing the risk of misclassification.

Reply: The overlap between heavy passive exposure and light active smoking may occur when using urinary cotinine concentrations alone, particularly at lower cutoff thresholds. As shown in the ROC analysis, specificity increases with higher cutoff values, which helps reduce potential misclassification between these exposure categories. The selected 7 ng/mL cutoff was chosen to balance sensitivity and specificity while minimizing misclassification risk; however, we recognize that biochemical measures cannot always fully distinguish heavy second-hand exposure from light active smoking. This limitation has now been explicitly acknowledged in the revised manuscript, and we emphasize that cotinine levels should be interpreted alongside self-reported smoking behavior and exposure history for more accurate classification.

7. Participants were recruited exclusively from hospital staff, patients, and university students. This convenience sampling frame does not reflect the general Saudi female population and introduces selection bias, limiting external validity. The manuscript does not acknowledge this limitation and incorrectly implies random sampling. This should be corrected and discussed.

Reply: The Study Population and Setting section has been revised to clearly specify the recruitment locations and timeframe. Participants were recruited between May and December 2024 from King Saud Medical City (including patients attending the sample collection department), employees at King Khalid University Hospital, and students from various colleges of King Saud University (KSU). The inclusion criteria are now explicitly stated: Saudi females aged ≥18 years who agreed to participate, were able to complete the questionnaire, and provided a urine sample. These revisions improve clarity regarding the study setting, recruitment strategy, and eligibility criteria, thereby enhancing transparency and reproducibility. A limitation sentence was added to the conclusion “A key limitation of this study was the difficulty in recruiting female participants for urine sample collection due to cultural and social sensitivities, which reduced the sample size and may limit generalizability.”

8. Several paragraphs in the Introduction provide extensive background on metabolomics and LC-MS/MS applications. While informative, some of this material does not directly advance the specific rationale of the present study. Streamlining this section to focus more clearly on the public health motivation and the application of LC-MS/MS for cotinine quantification would improve clarity.

Reply: The introduction paragraph on LC-MS/MS application was revised to focus on focus the public health relevance of tobacco exposure assessment and the specific application of LC-MS/MS for urinary cotinine quantification.

9. The study collected a rich set of demographic and behavioral variables. However, the statistical analysis is largely descriptive. Conducting multivariable analyses to assess independent predictors of urinary cotinine levels or smoking status would add depth to the results and enhance the overall robustness of the conclusions.

Reply: We agree that multivariable analysis could provide additional insight into independent predictors of urinary cotinine levels or smoking status. However, the primary objective of this study was methodological namely, the validation of an LC-MS/MS method and the establishment of a population-specific cutoff value for urinary cotinine. Additionally, the relatively small sample size and the limited number of confirmed smokers reduced the statistical power required to perform reliable multivariable modeling without risking model overfitting and unstable estimates. Therefore, the analysis was intentionally focused on descriptive statistics and ROC-based diagnostic evaluation, which were aligned with the study objectives.

10. The manuscript would benefit from clarification on whether the questionnaire was pilot-tested or validated prior to use. If these steps were undertaken, they should be reported. If not, acknowledging this as a limitation would improve transparency.

Reply: The questionnaire was specifically developed to align with the objectives of the present study and was structured into nine clearly defined sections covering demographic characteristics, smoking status, smoking history, frequency and duration, quit attempts, second-hand smoke exposure, health impacts, and open-ended feedback. The questionnaire was reviewed internally by the research team to ensure clarity, relevance, and content appropriateness prior to implementation. However, a formal psychometric validation process was not conducted. We have also acknowledged this as a methodological limitation in the conclusion section.

11. Urinary cotinine concentrations exhibit marked skewness and substantial divergence between means and medians (Table 2). Although group comparisons were performed using unpaired t-tests, variance tests indicate significant heteroscedasticity (Table 3). In this context, alternative approaches that do not assume equal variances (e.g., Welch’s t-test) or nonparametric or transformed analyses would provide more robust support for the reported group differences.

Reply: Due to the study’s sample size and scope, we did not re-run alternative statistical analyses; however, the results remain descriptive and intended primarily to illustrate patterns in cotinine levels across groups. We have now acknowledged this limitation in the revised manuscript and recommend that future studies with larger cohorts consider Welch’s t-test, nonparametric tests, or data transformation to strengthen statistical robustness.

12. The presence of extreme values among smokers and passive smokers suggests that some group differences may be influenced by a small number of high observations. Complementary median-based or nonparametric analyses would improve robustness.

Reply: We have now acknowledged in the revised manuscript that extreme values and skewed distributions may affect mean-based comparisons, and we recommend that future studies with larger sample sizes incorporate median-based or nonparametric analyses to enhance statistical robustness.

13. Because vaping is reported among participants, and nicotine delivery differs from combustible tobacco use, failure to account for vaping behavior may confound cotinine-based classification. This should be addressed analytically or acknowledged as a limitation.

Reply: We acknowledge that vaping was reported among some participants and that nicotine delivery from electronic cigarettes may differ in pattern and intensity compared with combustible tobacco products. Because urinary cotinine reflects systemic nicotine exposure regardless of the source, it does not distinguish between combustible tobacco use and vaping. The primary aim of this study was to assess overall nicotine exposure using cotinine as a biomarker rather than to differentiate between nicotine delivery methods. However, we agree that the presence of vaping may introduce heterogeneity in exposure patterns and could potentially influence cotinine-based classification. This has now been explicitly acknowledged as a limitation in the revised manuscript. Future studies with larger cohorts should stratify analyses by product type (combustible vs. electronic) to better characterize exposure dynamics.

14. Figure 4 is described as part of the LC-MS optimization. However, its contribution to the main quantitative and exposure analyses is limited. The authors may consider justifying its inclusion as a main figure or moving it to the Supplementary Information.

Reply: Figure 4 was moved to supplementary.

15. With only 15 self-reported smokers, the study has limited power to analyze smoking behaviors (Objective 4), and the ROC-based cutoff determination is statistically limited. This limitation should be explicitly acknowledged and conclusions tempered accordingly.

Reply: We acknowledge that the relatively small number of self-reported smokers (n = 15) limits statistical power for detailed analysis of smoking behaviors (Objective 4) and may affect the stability and precision of the ROC-based cutoff determination. The primary aim of the study was methodological, focusing on biomarker validation and preliminary cutoff estimation within this specific population. We agree that the limited number of smokers constrains subgroup analyses and may influence the robustness of diagnostic performance estimates. This limitation has now been explicitly acknowledged in the revised manuscript, and the conclusions have been tempered to reflect that the proposed cutoff should be considered preliminary and requires confirmation in larger cohorts.

Minor comments

Urinary cotinine concentrations are reported without correction for urine dilution (e.g., creatinine normalization). This should be justified or acknowledged as a limitation.

Reply: We acknowledge that urinary cotinine concentrations were not corrected for urine dilution using creatinine normalization. This has now been explicitly stated in the manuscript as a limitation, and we note that future studies should consider creatinine-adjusted cotinine measurements to improve accuracy and comparability.

The timing of urine collection relative to last nicotine exposure is not described and may influence cotinine levels. Therefore, clarification would improve interpretability.

Reply: We agree that the timing of urine collection relative to last nicotine exposure may influence cotinine concentrations. Since the exact timing was not systematically recorded, this has now been clarified and acknowledged as a limitation in the revised manuscript.

The criteria used to classify participants as “passive smokers” are not clearly defined and should be expl

Attachment Submitted filename: Response to Reviewers.docx

# Peer review: Decision Letter 1

**DOI:** 10.1371/journal.pone.0355166.r003

**Authors:** Thiago Fernandes

18 May 2026

PONE-D-25-62265R1

Biomarker-based Assessment of Tobacco Smoke Exposure in Saudi Females via analysis of Urinary Cotinine level

PLOS One

Dear Dr. Alanazi,

Thank you for submitting your manuscript to PLOS ONE. After careful consideration, we feel that it has merit but does not fully meet PLOS ONE’s publication criteria as it currently stands. Therefore, we invite you to submit a revised version of the manuscript that addresses the points raised during the review process.

==============================

Please respond to all comments and highlight the changes in the revised ms.

==============================

Please submit your revised manuscript by Jul 02 2026 11:59PM. If you will need more time than this to complete your revisions, please reply to this message or contact the journal office at plosone@plos.org. When you're ready to submit your revision, log on to https://www.editorialmanager.com/pone/ and select the 'Submissions Needing Revision' folder to locate your manuscript file.

Please include the following items when submitting your revised manuscript:

- A letter that responds to each point raised by the academic editor and reviewer(s). You should upload this letter as a separate file labeled 'Response to Reviewers'.
- A marked-up copy of your manuscript that highlights changes made to the original version. You should upload this as a separate file labeled 'Revised Manuscript with Track Changes'.
- An unmarked version of your revised paper without tracked changes. You should upload this as a separate file labeled 'Manuscript'.

If you would like to make changes to your financial disclosure, please include your updated statement in your cover letter. Guidelines for resubmitting your figure files are available below the reviewer comments at the end of this letter.

If applicable, we recommend that you deposit your laboratory protocols in protocols.io to enhance the reproducibility of your results. Protocols.io assigns your protocol its own identifier (DOI) so that it can be cited independently in the future. For instructions see: https://journals.plos.org/plosone/s/submission-guidelines#loc-laboratory-protocols. Additionally, PLOS ONE offers an option for publishing peer-reviewed Lab Protocol articles, which describe protocols hosted on protocols.io. Read more information on sharing protocols at https://plos.org/protocols?utm_medium=editorial-email&utm_source=authorletters&utm_campaign=protocols.

As the corresponding author, your ORCID iD is verified in the submission system and will appear in the published article. PLOS supports the use of ORCID, and we encourage all coauthors to register for an ORCID iD and use it as well. Please encourage your coauthors to verify their ORCID iD within the submission system before final acceptance, as unverified ORCID iDs will not appear in the published article. *Only*  the individual author can complete the verification step; PLOS staff *cannot*  verify ORCID iDs on behalf of authors.

We look forward to receiving your revised manuscript.

Kind regards,

Thiago P. Fernandes, PhD

Academic Editor

PLOS One

Journal Requirements:

If the reviewer comments include a recommendation to cite specific previously published works, please review and evaluate these publications to determine whether they are relevant and should be cited. There is no requirement to cite these works unless the editor has indicated otherwise.

[Note: HTML markup is below. Please do not edit.]

Reviewers' comments:

Reviewer's Responses to Questions

**Comments to the Author**

1. If the authors have adequately addressed your comments raised in a previous round of review and you feel that this manuscript is now acceptable for publication, you may indicate that here to bypass the “Comments to the Author” section, enter your conflict of interest statement in the “Confidential to Editor” section, and submit your "Accept" recommendation.

Reviewer #1: (No Response)

Reviewer #3: (No Response)

**********

2. Is the manuscript technically sound, and do the data support the conclusions?

The manuscript must describe a technically sound piece of scientific research with data that supports the conclusions. Experiments must have been conducted rigorously, with appropriate controls, replication, and sample sizes. The conclusions must be drawn appropriately based on the data presented.

Reviewer #1: Partly

Reviewer #3: Partly

**********

3. Has the statistical analysis been performed appropriately and rigorously?

Reviewer #1: No

Reviewer #3: No

**********

4. Have the authors made all data underlying the findings in their manuscript fully available?

The PLOS Data policy requires authors to make all data underlying the findings described in their manuscript fully available without restriction, with rare exception (please refer to the Data Availability Statement in the manuscript PDF file). The data should be provided as part of the manuscript or its supporting information, or deposited to a public repository. For example, in addition to summary statistics, the data points behind means, medians and variance measures should be available. If there are restrictions on publicly sharing data—e.g. participant privacy or use of data from a third party—those must be specified.

Reviewer #1: Yes

Reviewer #3: Yes

**********

5. Is the manuscript presented in an intelligible fashion and written in standard English?

PLOS ONE does not copyedit accepted manuscripts, so the language in submitted articles must be clear, correct, and unambiguous. Any typographical or grammatical errors should be corrected at revision, so please note any specific errors here.

Reviewer #1: Yes

Reviewer #3: Yes

**********

6. Review Comments to the Author

Please use the space provided to explain your answers to the questions above. You may also include additional comments for the author, including concerns about dual publication, research ethics, or publication ethics. (Please upload your review as an attachment if it exceeds 20,000 characters)

Reviewer #1: Most of the previous comments have been addressed in the revised manuscript. However, several issues remain only partially resolved and would benefit from further clarification or revision:

Major point:

The authors indicate that alternative statistical analyses were not performed due to the study’s sample size and scope. However, the concern raised relates to the appropriateness of the statistical test used rather than the study scope. Because variance tests indicate significant heteroscedasticity (p < 0.0001), the assumptions of the standard unpaired t-test are violated. Under these conditions, a Welch’s t-test or a nonparametric test (e.g., Mann–Whitney) would be more appropriate. Simply acknowledging this as a limitation does not fully resolve the methodological concern, and small sample size does not justify the use of a statistical test whose assumptions are not satisfied. Because these statistical comparisons directly support the reported results, the choice of statistical test may influence the strength of the study’s conclusions. As such, the currently reported p-values may not be optimal.

Minor points:

1. Given the limited sample size and the convenience sampling design, the study appears exploratory in nature. The manuscript would benefit from clarifying whether it should be framed as a pilot or exploratory study and from tempering statements suggesting the establishment of a population-specific cutoff. The need for external validation in larger and more representative cohorts should be emphasized.

2. The ROC analysis should report the Area Under the Curve (AUC) with its 95% confidence interval, as these are standard measures of diagnostic performance.

Example:

• Area under the ROC curve (AUC) = 0.94

• 95% CI = 0.88–0.99

• p < 0.001

3. Given the pronounced skewness and the presence of extreme values in urinary cotinine concentrations (Table 2 and Figure 5), mean-based comparisons may not fully represent the underlying distribution. Reporting median and interquartile range (IQR) values and/or performing nonparametric statistical analyses (e.g., Mann–Whitney test) would provide a more robust comparison between groups. Acknowledging this only as a future direction does not fully address this.

4. Because the ROC analysis is based on a relatively small number of smokers, the interpretation of diagnostic performance should be moderated. Describing the results as demonstrating “excellent diagnostic performance” may be overly strong. The proposed cutoff may be better described as preliminary and requiring validation in larger cohorts.

Reviewer #3: Thank you for the opportunity to review the manuscript. I consider the topic relevant and recognize the research's potential to contribute to the scientific development of the field. However, some methodological issues deserve attention, as highlighted below:

At certain points, the text becomes verbose and confusing. For example, at the end of the introduction, the authors justify the research and present the objective repetitively, reiterating this information more than once. It is suggested that the introduction be reorganized in a funnel format, starting with more general aspects of the topic, moving on to more specific elements, and ending with a clear presentation of the study's objective and hypotheses. Therefore, a structural review of the text is recommended to make it more concise, fluid, and objective.

In the methods section, it is suggested that the authors provide more detailed information about the procedures adopted in the study. It is recommended to specify the location where the research was conducted, the laboratory or institution responsible for analyzing the samples, as well as the professionals involved in collecting and evaluating these samples. Furthermore, it would be important to clarify how the recruitment sites were defined, indicating whether the selection occurred by convenience or through other methodological criteria. A clearer description of the study population is also suggested, including its main characteristics and eligibility criteria, in order to facilitate understanding of the methodological design and the reproducibility of the research.

In the section regarding sample size calculation, the authors report that it was not possible to reach the estimated sample size. However, in a previous section, the statement remains that the data collection was conducted until the N suggested by the sample size calculation was reached, which generates inconsistency in the methodological description. It is suggested that this information be reviewed and harmonized to ensure greater clarity and coherence in the text.

It is also suggested that the authors describe the sample recruitment process in more detail, clarifying, for example, whether the selection occurred by convenience and how the participants had access to information about the research. It is important to specify how these women were recruited, what dissemination strategies were used, and in what contexts the participants were recruited. Furthermore, some of the highlighted information is scattered throughout the text, in sections that do not adequately correspond to the content presented. It is recommended to reorganize this information into a single appropriate topic or subsection, in order to promote clarity, methodological organization, and reader comprehension.

The last sentence of the section regarding the sample size calculation was unclear and, as written, compromises the understanding of the passage. It is suggested that its wording be revised or relocated to another section of the manuscript where the information can be better contextualized. It is also recommended that the authors provide more detailed information about the questionnaire used in the study, specifying whether the instrument underwent a validation or adaptation process or whether it was developed based on previously existing scientific literature. This information is important to strengthen the methodological quality and reliability of the data obtained.

Furthermore, the methodological description implied that the Informed Consent Form was included as part of the questionnaire. If this interpretation is correct, clarification is necessary, since, ethically, participants must have access to the ICF before the start of the research and prior to submitting to any data collection instrument. It is suggested that the authors review the description of this procedure to avoid ambiguities and ensure greater clarity regarding the ethical aspects of the study.

In my perception, the description of the sampling technique should be included in the section referring to the sample, since it is information directly related to the composition and selection of the study participants. As it is currently presented, after the description of the instruments, the information becomes decontextualized and loses its methodological function. This aspect reinforces the perception of structural disorganization in the text, highlighting the need for revision and reorganization of the methodological sections, in order to promote greater clarity, coherence, and fluency in reading the manuscript. Throughout the reading of the manuscript, after the description of the instruments used, I felt the lack of a more detailed explanation about the methods employed for the evaluation of urine samples. Later, I realized that this information is described in another section of the text and in an appropriate way. However, it is suggested that this information be reorganized, positioning it immediately after the instruments section, since it refers directly to the techniques and procedures for data collection.

At times, the writing of the manuscript presents characteristics closer to a research project than to a scientific article proper. As an example, the inclusion of extensive information on the risks and benefits of the research stands out, an aspect that, depending on the journal's guidelines, may require a more objective and synthetic approach.

In addition, if the authors choose to maintain the description of the risks involved, greater methodological detail is suggested. The text states that the research offered minimal risks to the participants, but does not specify what these risks would be, nor does it describe the strategies adopted to minimize them. It is recommended to clarify these aspects in order to give greater ethical and methodological consistency to the manuscript.

On line 267, the authors reiterate the study's objective, information that had already been clearly presented at the end of the introduction. This repetition can also be observed at other points in the manuscript, contributing to redundant and less fluid writing. Therefore, a general review of the writing is suggested, in order to make the text more objective, cohesive, and concise, avoiding unnecessary repetitions throughout the sections.

It is suggested that the classification of smoking status be presented before the topic concerning data integration. This is because the data integration section establishes a more direct connection with the data analysis stage and should therefore be positioned in logical sequence to the analyses performed. The current organization compromises methodological fluidity and makes it difficult to understand the structure of the study design. It is recommended to reorganize these subsections in order to favor greater coherence and chaining of the information presented.

It was not clear in the manuscript whether the analysis of the data distribution was performed prior to the application of statistical tests. Considering that parametric tests, such as ANOVA and t-tests, were used, it is suggested that the authors report whether the assumptions necessary for these analyses were verified, especially regarding the normality of the data distribution and the homogeneity of variances. If these analyses were conducted, it is recommended to describe the procedures adopted and the tests used for this verification.

In the discussion section, I felt the lack of a more in-depth articulation with the literature in the area, especially in the sense of comparing the study's findings with previously published evidence, corroborating or contradicting them when pertinent. The inclusion of this discussion is important to strengthen the interpretation of the results and situate the study's contribution in the scientific field.

In addition, it is considered that the limitations section would be more appropriately positioned at the end of the discussion, and not in the conclusion. The conclusion should prioritize the synthesis of the main findings and implications of the study, avoiding the insertion of new analytical elements.

Finally, it is suggested that, in addition to describing the limitations of the research, the authors also clarify which methodological strategies were adopted to minimize its potential impacts. This discussion can strengthen the robustness of the study and demonstrate greater scientific rigor in conducting the research.

**********

7. PLOS authors have the option to publish the peer review history of their article (what does this mean?). If published, this will include your full peer review and any attached files.

If you choose “no”, your identity will remain anonymous but your review may still be made public.

**Do you want your identity to be public for this peer review?**  For information about this choice, including consent withdrawal, please see our Privacy Policy.

Reviewer #1: No

Reviewer #3: No

**********

[NOTE: If reviewer comments were submitted as an attachment file, they will be attached to this email and accessible via the submission site. Please log into your account, locate the manuscript record, and check for the action link "View Attachments". If this link does not appear, there are no attachment files.]

To ensure your figures meet our technical requirements, please review our figure guidelines: https://journals.plos.org/plosone/s/figures

You may also use PLOS’s free figure tool, NAAS, to help you prepare publication quality figures: https://journals.plos.org/plosone/s/figures#loc-tools-for-figure-preparation.

NAAS will assess whether your figures meet our technical requirements by comparing each figure against our figure specifications.

# Author comment: Author response to Decision Letter 2

**DOI:** 10.1371/journal.pone.0355166.r004

25 May 2026

Author Reply List

To

Prof. Thiago P. Fernandes, PhD

Academic Editor

PLOS One

First thanks for handling our manuscript and considering it for publication in PLOS ONE after careful revision. Kindly, find all our responses to the raised queries. The manuscript has been carefully revised.

Review Comments to the Author

Reviewer #1: Most of the previous comments have been addressed in the revised manuscript. However, several issues remain only partially resolved and would benefit from further clarification or revision:

Reply: Thanks a lot for the reviewer comments, we appreciate all the efforts made for the evaluation of our manuscript. The manuscript was revised and the raised points were addressed and we hope the updated version fulfils the reviewer’s requirements.

Major point:

The authors indicate that alternative statistical analyses were not performed due to the study’s sample size and scope. However, the concern raised relates to the appropriateness of the statistical test used rather than the study scope. Because variance tests indicate significant heteroscedasticity (p < 0.0001), the assumptions of the standard unpaired t-test are violated. Under these conditions, a Welch’s t-test or a nonparametric test (e.g., Mann–Whitney) would be more appropriate. Simply acknowledging this as a limitation does not fully resolve the methodological concern, and small sample size does not justify the use of a statistical test whose assumptions are not satisfied. Because these statistical comparisons directly support the reported results, the choice of statistical test may influence the strength of the study’s conclusions. As such, the currently reported p-values may not be optimal.

Reply: Thanks a lot for the reviewer’s comment, , the statistical analyses were revised to account for the significant heteroscedasticity observed in urinary cotinine concentrations. Brown–Forsythe and Bartlett’s tests confirmed unequal variances across smoking categories (p < 0.0001). Therefore, pairwise comparisons were re-evaluated using Welch’s corrected unpaired t-tests, which do not assume equal variances. Median and interquartile range (IQR) values were also added to better represent the skewed distribution and influence of extreme observations. The revised analyses supported the original interpretation while providing a more statistically robust assessment of group differences.

Minor points:

1. Given the limited sample size and the convenience sampling design, the study appears exploratory in nature. The manuscript would benefit from clarifying whether it should be framed as a pilot or exploratory study and from tempering statements suggesting the establishment of a population-specific cutoff. The need for external validation in larger and more representative cohorts should be emphasized.

Reply: The manuscript has been revised to clarify that the present work should be considered an exploratory/pilot biomarker study due to the modest sample size and convenience sampling design. Statements implying definitive establishment of a population-specific cutoff have been moderated throughout the manuscript. The proposed 7 ng/mL threshold is now described as a preliminary cutoff requiring validation in larger and more representative cohorts.

2. The ROC analysis should report the Area Under the Curve (AUC) with its 95% confidence interval, as these are standard measures of diagnostic performance.

Example:

• Area under the ROC curve (AUC) = 0.94

• 95% CI = 0.88–0.99

• p < 0.001

Reply: The ROC analysis section has been revised to include the Area Under the Curve (AUC), corresponding 95% confidence interval, and p-value as standard indicators of diagnostic performance. These values are now explicitly reported in both the Results section and the figure legend for improved transparency and interpretability.

3. Given the pronounced skewness and the presence of extreme values in urinary cotinine concentrations (Table 2 and Figure 5), mean-based comparisons may not fully represent the underlying distribution. Reporting median and interquartile range (IQR) values and/or performing nonparametric statistical analyses (e.g., Mann–Whitney test) would provide a more robust comparison between groups. Acknowledging this only as a future direction does not fully address this.

Reply: We appreciate the reviewer’s suggestion, median and interquartile range (IQR) values for urinary cotinine concentrations have now been added to the Results section and relevant tables to better represent the skewed distribution of the data.

4. Because the ROC analysis is based on a relatively small number of smokers, the interpretation of diagnostic performance should be moderated. Describing the results as demonstrating “excellent diagnostic performance” may be overly strong. The proposed cutoff may be better described as preliminary and requiring validation in larger cohorts.

Reply: The wording describing the ROC performance has been revised and moderated throughout the manuscript. Rather than describing the diagnostic performance as “excellent,” the manuscript now states that the ROC analysis demonstrated “good preliminary discriminatory performance.” We also emphasize that the proposed cutoff value should be considered preliminary and requires external validation in larger and more representative populations.

Reviewer #3: Thank you for the opportunity to review the manuscript. I consider the topic relevant and recognize the research's potential to contribute to the scientific development of the field. However, some methodological issues deserve attention, as highlighted below:

Reply: Thanks a lot for the reviewer comments, we appreciate all the efforts made for the evaluation of our manuscript. The manuscript was revised and the raised points were addressed, and we hope the updated version fulfils the reviewer’s requirements.

At certain points, the text becomes verbose and confusing. For example, at the end of the introduction, the authors justify the research and present the objective repetitively, reiterating this information more than once. It is suggested that the introduction be reorganized in a funnel format, starting with more general aspects of the topic, moving on to more specific elements, and ending with a clear presentation of the study's objective and hypotheses. Therefore, a structural review of the text is recommended to make it more concise, fluid, and objective.

Reply: The Introduction has been substantially reorganized following a funnel structure, beginning with the broader public health significance of tobacco exposure, followed by the role of urinary cotinine and LC-MS/MS analysis, and concluding with the specific rationale and objectives of the present study.

In the methods section, it is suggested that the authors provide more detailed information about the procedures adopted in the study. It is recommended to specify the location where the research was conducted, the laboratory or institution responsible for analyzing the samples, as well as the professionals involved in collecting and evaluating these samples. Furthermore, it would be important to clarify how the recruitment sites were defined, indicating whether the selection occurred by convenience or through other methodological criteria. A clearer description of the study population is also suggested, including its main characteristics and eligibility criteria, in order to facilitate understanding of the methodological design and the reproducibility of the research.

Reply: We appreciate the reviewer’s recommendation. The Methods section has been revised to provide more detailed information regarding the study setting, recruitment process, eligibility criteria, and sample collection procedures. The manuscript now clearly states that participants were recruited by convenience sampling from King Saud Medical City, King Khalid University Hospital employees, and students from King Saud University between May and December 2024. Additional details regarding the laboratory analysis setting and urine sample processing procedures were also added to improve reproducibility and methodological transparency.

In the section regarding sample size calculation, the authors report that it was not possible to reach the estimated sample size. However, in a previous section, the statement remains that the data collection was conducted until the N suggested by the sample size calculation was reached, which generates inconsistency in the methodological description. It is suggested that this information be reviewed and harmonized to ensure greater clarity and coherence in the text.

Reply: We thank the reviewer for identifying this inconsistency. The sample size calculation section has been revised and harmonized throughout the manuscript. The revised text now clearly explains that although the calculated target sample size was 385 participants, recruitment challenges and eligibility criteria resulted in a final analyzable sample of 133 participants. Statements implying that the target sample size was fully achieved were removed for consistency and clarity.

It is also suggested that the authors describe the sample recruitment process in more detail, clarifying, for example, whether the selection occurred by convenience and how the participants had access to information about the research. It is important to specify how these women were recruited, what dissemination strategies were used, and in what contexts the participants were recruited. Furthermore, some of the highlighted information is scattered throughout the text, in sections that do not adequately correspond to the content presented. It is recommended to reorganize this information into a single appropriate topic or subsection, in order to promote clarity, methodological organization, and reader comprehension.

Reply: The Sampling Technique subsection has been revised to clarify that convenience sampling was employed. Additional details describing recruitment procedures and dissemination strategies have also been added. Eligible participants from hospitals and universities in Riyadh were approached directly and invited to participate voluntarily after receiving information regarding the study objectives and procedures.

The last sentence of the section regarding the sample size calculation was unclear and, as written, compromises the understanding of the passage. It is suggested that its wording be revised or relocated to another section of the manuscript where the information can be better contextualized.

Reply: The sample size section has been revised and simplified to improve clarity and contextual coherence. The sentence referring to the Saudi female population size was rewritten and integrated appropriately within the sample size rationale.

It is also recommended that the authors provide more detailed information about the questionnaire used in the study, specifying whether the instrument underwent a validation or adaptation process or whether it was developed based on previously existing scientific literature. This information is important to strengthen the methodological quality and reliability of the data obtained.

Reply: Additional information regarding questionnaire development has been incorporated into the revised manuscript. The questionnaire was specifically designed to address the objectives of the study based on relevant literature and smoking exposure variables. The manuscript now also clarifies that, although the questionnaire was reviewed for clarity and relevance, it did not undergo formal psychometric validation, which has been acknowledged as a study limitation.

Furthermore, the methodological description implied that the Informed Consent Form was included as part of the questionnaire. If this interpretation is correct, clarification is necessary, since, ethically, participants must have access to the ICF before the start of the research and prior to submitting to any data collection instrument. It is suggested that the authors review the description of this procedure to avoid ambiguities and ensure greater clarity regarding the ethical aspects of the study.

Reply: The Ethical Considerations section has been revised to clarify that participants were first provided with a detailed participant information sheet explaining the study objectives, procedures, confidentiality measures, risks, and benefits before participation. Electronic written informed consent was obtained prior to questionnaire completion and urine sample collection. The revised wording eliminates ambiguity regarding the consent process.

In my perception, the description of the sampling technique should be included in the section referring to the sample, since it is information directly related to the composition and selection of the study participants. As it is currently presented, after the description of the instruments, the information becomes decontextualized and loses its methodological function. This aspect reinforces the perception of structural disorganization in the text, highlighting the need for revision and reorganization of the methodological sections, in order to promote greater clarity, coherence, and fluency in reading the manuscript. Throughout the reading of the manuscript, after the description of the instruments used, I felt the lack of a more detailed explanation about the methods employed for the evaluation of urine samples. Later, I realized that this information is described in another section of the text and in an appropriate way. However, it is suggested that this information be reorganized, positioning it immediately after the instruments section, since it refers directly to the techniques and procedures for data collection.

Reply: The Methods section has been reorganized to improve logical flow. The urine sample preparation and LC-MS/MS analytical procedures are now positioned directly after the questionnaire and sampling descriptions, facilitating better methodological continuity.

At times, the writing of the manuscript presents characteristics closer to a research project than to a scientific article proper. As an example, the inclusion of extensive information on the risks and benefits of the research stands out, an aspect that, depending on the journal's guidelines, may require a more objective and synthetic approach. In addition, if the authors choose to maintain the description of the risks involved, greater methodological detail is suggested. The text states that the research offered minimal risks to the participants, but does not specify what these risks would be, nor does it describe the strategies adopted to minimize them. It is recommended to clarify these aspects in order to give greater ethical and methodological consistency to the manuscript.

Reply: The manuscript has undergone substantial language and structural editing to improve conciseness and align the presentation with standard scientific article formatting. Excessively detailed descriptions regarding risks and benefits were shortened and streamlined. The Ethical Considerations section has been revised to clarify that the study involved minimal risk due to non-invasive urine sample collection and completion of a self-administered questionnaire. Confidentiality protections, anonymization procedures, and voluntary participation rights are now described more clearly to demonstrate the measures adopted to minimize potential risks.

On line 267, the authors reiterate the study's objective, information that had already been clearly presented at the end of the introduction. This repetition can also be observed at other points in the manuscript, contributing to redundant and less fluid writing. Therefore, a general review of the writing is suggested, in order to make the text more objective, cohesive, and concise, avoiding unnecessary repetitions throughout the sections.

Reply: Repeated statements of the study objectives throughout the manuscript have been removed or condensed. The manuscript was carefully revised to improve cohesion, reduce redundancy, and enhance readability.

It is suggested that the classification of smoking status be presented before the topic concerning data integration. This is because the data integration section establishes a more direct connection with the data analysis stage

Attachment Submitted filename: Response_to_Reviewers_auresp_2.docx

# Peer review: Decision Letter 2

**DOI:** 10.1371/journal.pone.0355166.r005

**Authors:** Thiago Fernandes

12 Jul 2026

<div>PONE-D-25-62265R2

Biomarker-based Assessment of Tobacco Smoke Exposure in Saudi Females via analysis of Urinary Cotinine level

PLOS One

Dear Dr. Alanazi,

Thank you for submitting your manuscript to PLOS ONE. After careful consideration, we feel that it has merit but does not fully meet PLOS ONE’s publication criteria as it currently stands. Therefore, we invite you to submit a revised version of the manuscript that addresses the points raised during the review process.

==============================

Please respond all comments and highlight the changes in the revised ms.

==============================

Please submit your revised manuscript by Aug 26 2026 11:59PM. If you will need more time than this to complete your revisions, please reply to this message or contact the journal office at plosone@plos.org. When you're ready to submit your revision, log on to https://www.editorialmanager.com/pone/ and select the 'Submissions Needing Revision' folder to locate your manuscript file.

Please include the following items when submitting your revised manuscript:

- A letter that responds to each point raised by the academic editor and reviewer(s). You should upload this letter as a separate file labeled 'Response to Reviewers'.
- A marked-up copy of your manuscript that highlights changes made to the original version. You should upload this as a separate file labeled 'Revised Manuscript with Track Changes'.
- An unmarked version of your revised paper without tracked changes. You should upload this as a separate file labeled 'Manuscript'.

If you would like to make changes to your financial disclosure, please include your updated statement in your cover letter. Guidelines for resubmitting your figure files are available below the reviewer comments at the end of this letter.

If applicable, we recommend that you deposit your laboratory protocols in protocols.io to enhance the reproducibility of your results. Protocols.io assigns your protocol its own identifier (DOI) so that it can be cited independently in the future. For instructions see: https://journals.plos.org/plosone/s/submission-guidelines#loc-laboratory-protocols. Additionally, PLOS ONE offers an option for publishing peer-reviewed Lab Protocol articles, which describe protocols hosted on protocols.io. Read more information on sharing protocols at https://plos.org/protocols?utm_medium=editorial-email&utm_source=authorletters&utm_campaign=protocols.

As the corresponding author, your ORCID iD is verified in the submission system and will appear in the published article. PLOS supports the use of ORCID, and we encourage all coauthors to register for an ORCID iD and use it as well. Please encourage your coauthors to verify their ORCID iD within the submission system before final acceptance, as unverified ORCID iDs will not appear in the published article. *Only*  the individual author can complete the verification step; PLOS staff *cannot*  verify ORCID iDs on behalf of authors.

We look forward to receiving your revised manuscript.

Kind regards,

Thiago P. Fernandes, PhD

Academic Editor

PLOS One

Journal Requirements:

If the reviewer comments include a recommendation to cite specific previously published works, please review and evaluate these publications to determine whether they are relevant and should be cited. There is no requirement to cite these works unless the editor has indicated otherwise.

Please review your reference list to ensure that it is complete and correct. If you have cited papers that have been retracted, please include the rationale for doing so in the manuscript text, or remove these references and replace them with relevant current references. Any changes to the reference list should be mentioned in the rebuttal letter that accompanies your revised manuscript. If you need to cite a retracted article, indicate the article’s retracted status in the References list and also include a citation and full reference for the retraction notice.

[Note: HTML markup is below. Please do not edit.]

Reviewers' comments:

Reviewer's Responses to Questions

**Comments to the Author**

1. If the authors have adequately addressed your comments raised in a previous round of review and you feel that this manuscript is now acceptable for publication, you may indicate that here to bypass the “Comments to the Author” section, enter your conflict of interest statement in the “Confidential to Editor” section, and submit your "Accept" recommendation.

Reviewer #1: (No Response)

Reviewer #3: All comments have been addressed

**********

2. Is the manuscript technically sound, and do the data support the conclusions?

The manuscript must describe a technically sound piece of scientific research with data that supports the conclusions. Experiments must have been conducted rigorously, with appropriate controls, replication, and sample sizes. The conclusions must be drawn appropriately based on the data presented.

Reviewer #1: Partly

Reviewer #3: (No Response)

**********

3. Has the statistical analysis been performed appropriately and rigorously?

Reviewer #1: No

Reviewer #3: (No Response)

**********

4. Have the authors made all data underlying the findings in their manuscript fully available?

The PLOS Data policy requires authors to make all data underlying the findings described in their manuscript fully available without restriction, with rare exception (please refer to the Data Availability Statement in the manuscript PDF file). The data should be provided as part of the manuscript or its supporting information, or deposited to a public repository. For example, in addition to summary statistics, the data points behind means, medians and variance measures should be available. If there are restrictions on publicly sharing data—e.g. participant privacy or use of data from a third party—those must be specified.

Reviewer #1: Yes

Reviewer #3: (No Response)

**********

5. Is the manuscript presented in an intelligible fashion and written in standard English?

PLOS ONE does not copyedit accepted manuscripts, so the language in submitted articles must be clear, correct, and unambiguous. Any typographical or grammatical errors should be corrected at revision, so please note any specific errors here.

Reviewer #1: Yes

Reviewer #3: (No Response)

**********

6. Review Comments to the Author

Please use the space provided to explain your answers to the questions above. You may also include additional comments for the author, including concerns about dual publication, research ethics, or publication ethics. (Please upload your review as an attachment if it exceeds 20,000 characters)

Reviewer #1: The authors have addressed several of the previous comments. However, the main statistical concern remains still only partially addressed.

Although the manuscript now states that Welch’s corrected unpaired t-tests were used, the values reported in Table 3 appear inconsistent with Welch’s test. The reported degrees of freedom, such as t(70), t(74), and t(116), correspond exactly to the standard equal-variance unpaired t-test calculation, n1 + n2 - 2. Since the variance tests showed significant heteroscedasticity, the authors should report the actual Welch-adjusted degrees of freedom, confidence intervals, and p-values.

The authors should also clarify the inconsistency in group sizes. The manuscript states that the refined classification included 11 smokers, 62 passive smokers, and 60 nonsmokers, whereas Tables 2 and 3 report 15 smokers, 61 passive smokers, and 57 nonsmokers. The classification used for each analysis should be clearly stated and applied consistently across the Results, tables, figures, and ROC analysis.

Finally, the authors should briefly explain how zero or non-detected cotinine values were handled in the logarithmic-scale plots.

Reviewer #3: (No Response)

**********

7. PLOS authors have the option to publish the peer review history of their article (what does this mean?). If published, this will include your full peer review and any attached files.

If you choose “no”, your identity will remain anonymous but your review may still be made public.

**Do you want your identity to be public for this peer review?**  For information about this choice, including consent withdrawal, please see our Privacy Policy.

Reviewer #1: No

Reviewer #3: No

**********

[NOTE: If reviewer comments were submitted as an attachment file, they will be attached to this email and accessible via the submission site. Please log into your account, locate the manuscript record, and check for the action link "View Attachments". If this link does not appear, there are no attachment files.]

To ensure your figures meet our technical requirements, please review our figure guidelines: https://journals.plos.org/plosone/s/figures

You may also use PLOS’s free figure tool, NAAS, to help you prepare publication quality figures: https://journals.plos.org/plosone/s/figures#loc-tools-for-figure-preparation.

NAAS will assess whether your figures meet our technical requirements by comparing each figure against our figure specifications.

# Author comment: Author response to Decision Letter 3

**DOI:** 10.1371/journal.pone.0355166.r006

14 Jul 2026

Author Reply List

To

Prof. Thiago P. Fernandes, PhD

Academic Editor

PLOS One

First thanks for handling our manuscript and considering it for publication in PLOS ONE after careful revision. Kindly, find all our responses to the raised queries. The manuscript has been carefully revised.

Review Comments to the Author

Reviewer #1: The authors have addressed several of the previous comments. However, the main statistical concern remains still only partially addressed.

Reply: Thanks a lot for the reviewers’ comments, we appreciate all the efforts made to the evaluation of our manuscript. We appreciate the efforts of the reviewer in the improvement of our manuscript. The manuscript was revised and the comments raised were addressed, and we hope the updated versions meet the reviewer requirements.

Although the manuscript now states that Welch’s corrected unpaired t-tests were used, the values reported in Table 3 appear inconsistent with Welch’s test. The reported degrees of freedom, such as t(70), t(74), and t(116), correspond exactly to the standard equal-variance unpaired t-test calculation, n1 + n2 - 2. Since the variance tests showed significant heteroscedasticity, the authors should report the actual Welch-adjusted degrees of freedom, confidence intervals, and p-values.

Reply: Thanks a lot for the reviewer’s comment, Table 3 was corrected to report the appropriate Welch-adjusted degrees of freedom, 95% confidence intervals, t statistics, and corresponding p-values generated by GraphPad Prism. These revisions do not alter the interpretation of the results, and the significant differences between smokers and the other study groups remain unchanged.

The authors should also clarify the inconsistency in group sizes. The manuscript states that the refined classification included 11 smokers, 62 passive smokers, and 60 nonsmokers, whereas Tables 2 and 3 report 15 smokers, 61 passive smokers, and 57 nonsmokers. The classification used for each analysis should be clearly stated and applied consistently across the Results, tables, figures, and ROC analysis.

Reply: The discrepancy resulted from presenting two different participant classifications. Specifically, the ROC analysis used the self-reported smoking status (15 self-reported smokers) as the reference classification, whereas the biomarker-based classification applied the urinary cotinine cutoff to categorize participants according to measured cotinine concentrations. The final statistical analyses were consistently performed using the complete study dataset comprising 15 smokers, 61 passive smokers, and 57 nonsmokers (n = 133). The manuscript has been carefully revised to ensure that these group sizes are reported consistently throughout the Results, Tables, Figures, and ROC analysis.

Finally, the authors should briefly explain how zero or non-detected cotinine values were handled in the logarithmic-scale plots.

Reply: A brief explanation has now been added to the Statistical Analysis section and to the figure 5 legend. Samples with non-detectable urinary cotinine concentrations were assigned a small positive value corresponding to the lower detection limit solely for visualization on logarithmic-scale graphs. This adjustment was applied only for graphical presentation and was not used in any statistical analyses, which were performed using the original measured values.

Attachment Submitted filename: Response_to_Reviewers_auresp_3.docx

# Editor report: Decision Letter 3

**DOI:** 10.1371/journal.pone.0355166.r007

**Authors:** Thiago Fernandes

20 Jul 2026

Biomarker-based Assessment of Tobacco Smoke Exposure in Saudi Females via analysis of Urinary Cotinine level

PONE-D-25-62265R3

Dear Dr. Alanazi,

We’re pleased to inform you that your manuscript has been judged scientifically suitable for publication and will be formally accepted for publication once it meets all outstanding technical requirements.

Within one week, you’ll receive an e-mail detailing the required amendments. When these have been addressed, you’ll receive a formal acceptance letter and your manuscript will be scheduled for publication.

An invoice will be generated when your article is formally accepted. Please note, if your institution has a publishing partnership with PLOS and your article meets the relevant criteria, all or part of your publication costs will be covered. Please make sure your user information is up-to-date by logging into Editorial Manager at Editorial Manager® and clicking the ‘Update My Information' link at the top of the page. For questions related to billing, please contact billing support.

If your institution or institutions have a press office, please notify them about your upcoming paper to help maximize its impact. If they’ll be preparing press materials, please inform our press team as soon as possible -- no later than 48 hours after receiving the formal acceptance. Your manuscript will remain under strict press embargo until 2 pm Eastern Time on the date of publication. For more information, please contact onepress@plos.org.

Kind regards,

Thiago P. Fernandes, PhD

Academic Editor

PLOS One

Additional Editor Comments (optional):

Please ensure during production that the participant classification applied to each analysis is described consistently throughout the manuscript (e.g. self-reported smoking status for the ROC analysis and biomarker-based classification), including the Results, Figs, and Tables. Wishing you success with the study.

Reviewers' comments:

# Editor report: Acceptance letter

**DOI:** 10.1371/journal.pone.0355166.r008

**Authors:** Thiago Fernandes

PONE-D-25-62265R3

PLOS One

Dear Dr. Alanazi,

I'm pleased to inform you that your manuscript has been deemed suitable for publication in PLOS One. Congratulations! Your manuscript is now being handed over to our production team.

At this stage, our production department will prepare your paper for publication. This includes ensuring the following:

* All references, tables, and figures are properly cited

* All relevant supporting information is included in the manuscript submission,

* There are no issues that prevent the paper from being properly typeset

You will receive further instructions from the production team, including instructions on how to review your proof when it is ready. Please keep in mind that we are working through a large volume of accepted articles, so please give us a few days to review your paper and let you know the next and final steps.

Lastly, if your institution or institutions have a press office, please let them know about your upcoming paper now to help maximize its impact. If they'll be preparing press materials, please inform our press team within the next 48 hours. Your manuscript will remain under strict press embargo until 2 pm Eastern Time on the date of publication. For more information, please contact onepress@plos.org.

You will receive an invoice from PLOS for your publication fee after your manuscript has reached the completed accept phase. If you receive an email requesting payment before acceptance or for any other service, this may be a phishing scheme. Learn how to identify phishing emails and protect your accounts at https://explore.plos.org/phishing.

If we can help with anything else, please email us at customercare@plos.org.

Thank you for submitting your work to PLOS ONE and supporting open access.

Kind regards,

PLOS ONE Editorial Office Staff

on behalf of

Dr. Thiago P. Fernandes

Academic Editor

PLOS One
